# Supplementary material for: Perineuronal nets decrease membrane capacitance of peritumoral fast spiking interneurons in a model of epilepsy
Source: Nat Commun. 2018 Nov 9;9:4724. doi: 10.1038/s41467-018-07113-0 (PMC6226462; doi:10.1038/s41467-018-07113-0)
Supplement: Supplementary file 1 — Supplementary Information [file 41467_2018_7113_MOESM1_ESM.pdf]

Supplementary Information

Perineuronal nets decrease membrane capacitance of peritumoral fast spiking interneurons in a model of epilepsy

Tewari *et. al*.

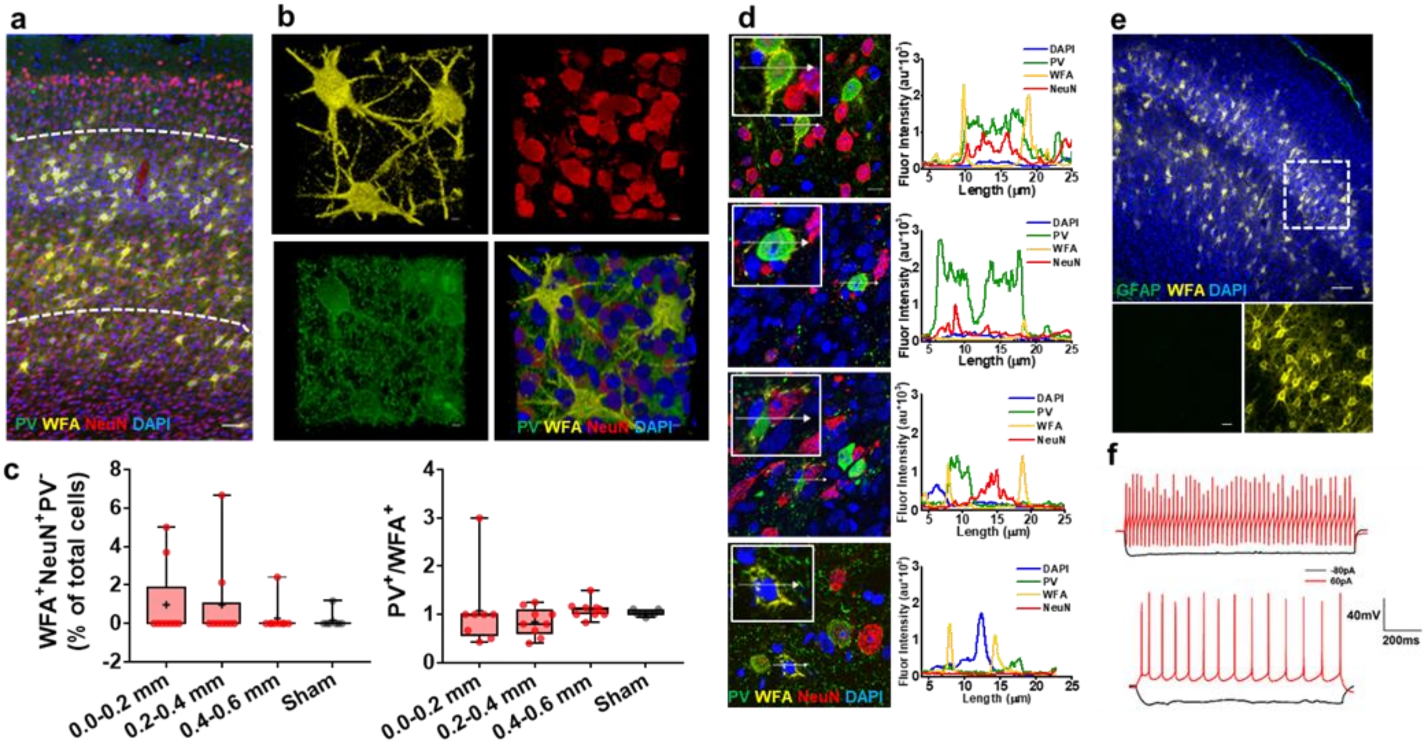

Supplementary Figure 1 PNNs in different experimental groups and FSNs spiking properties. **a, b** Immunofluorescence images of WFA (Yellow), NeuN (Red), PV (Green) and DAPI (blue) showing highest density of PNNs in cortical layers inner 2/3-5 (between dotted lines). 3D volume images (**b**) showing PNN around the soma and proximal dendrites of PV neurons. Scale=50 µm in (a) and 5 µm in (b)**. c** Percent of total cells showing $WFA^{+}NeuN^{+}PV^{-}$ (left), and PV^{+}/WFA^{+} ratio (right) did not show significant alterations in peritumoral brains and sham (One-way ANOVA, Sidak's posthoc test, n=7 sections from 4 mice in control, n=9 sections from 5 mice in PTC). **d** Representative images from the tumor border showing a range of neuronal morphologies and PNN disintegration. Top left image distal from the glioma border shows a PV^{+} interneuron with normal morphology and intact PNN. Top right - Line intensity profile of the same cell showing high intensity PV and NeuN fluorescence intensity flanked by WFA peaks that are confined to the cell membrane (Scale=10 µm). PV^{+} interneurons surrounded by glioma cells (dense DAPI-stained cells) in images on the left (second and third from top) are in the process of dying, show barely detectable PNNs; and the corresponding line intensity profiles are shown on the right (second and third from top). Dead "Ghost" cell (bottom left) shows no detectable PV and NeuN staining but remnants of PNN. Line profile (bottom right) shows WFA peaks, indicative of cell membrane containing high DAPI label binding to defragmented DNA. The cells used for line profile analysis are magnified in the inset images and line intensity profile indicates the fluorescence intensities along the line (arrow). Scale=10 µm. au – arbitrary units of fluorescence. **e** Representative immunofluorescence images from sham-injected mice showing no reactive astrogliosis (GFAP) and normal PNNs (WFA) cortex. Scale=50µm**. f** Representative characteristic spike firing pattern of PNN expressing fast spiking PV interneurons (FSNs) (top traces) and excitatory neurons (bottom traces) on same current injections.

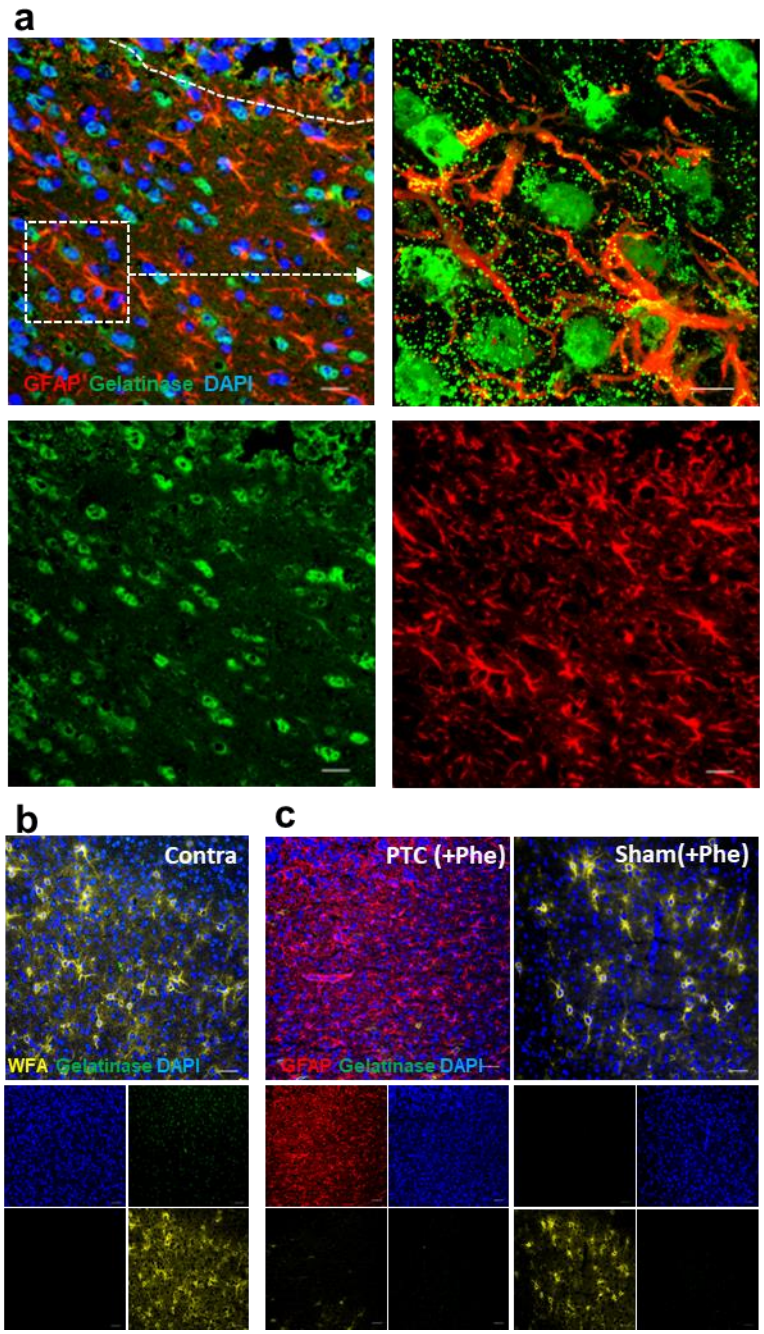

Supplementary Figure 2 Gelatinase activity in different experimental groups. **a** Representative immunofluorescence images of GFAP (Red) and gelatinase activity (Green) with DAPI (Blue) from peritumoral cortex (glioma border delimited by dotted line) showing the localization of gelatinase activity primarily in GFAP negative cells. Scale=20 µm. High magnification maximum intensity projection images of the dotted area in image (b) showed no appreciable co-localization of gelatinase activity and GFAP (top right). Scale=10 µm. **b** Contralateral cortex of GBM22 injected brain showing basal gelatinase activity (green), intact PNNs (WFA) and no reactive astrogliosis evident by unaltered GFAP expression (red). Scale=50 µm. **c** Pre-incubation of sections with the gelatinase inhibitor 1,10- phenanthroline (Phe) blocked the gelatinase activity in peritumoral and sham cortical tissues as evident by no apparent fluorescein fluorescence (Green) thereby serving as a negative control. Scale=50 µm.

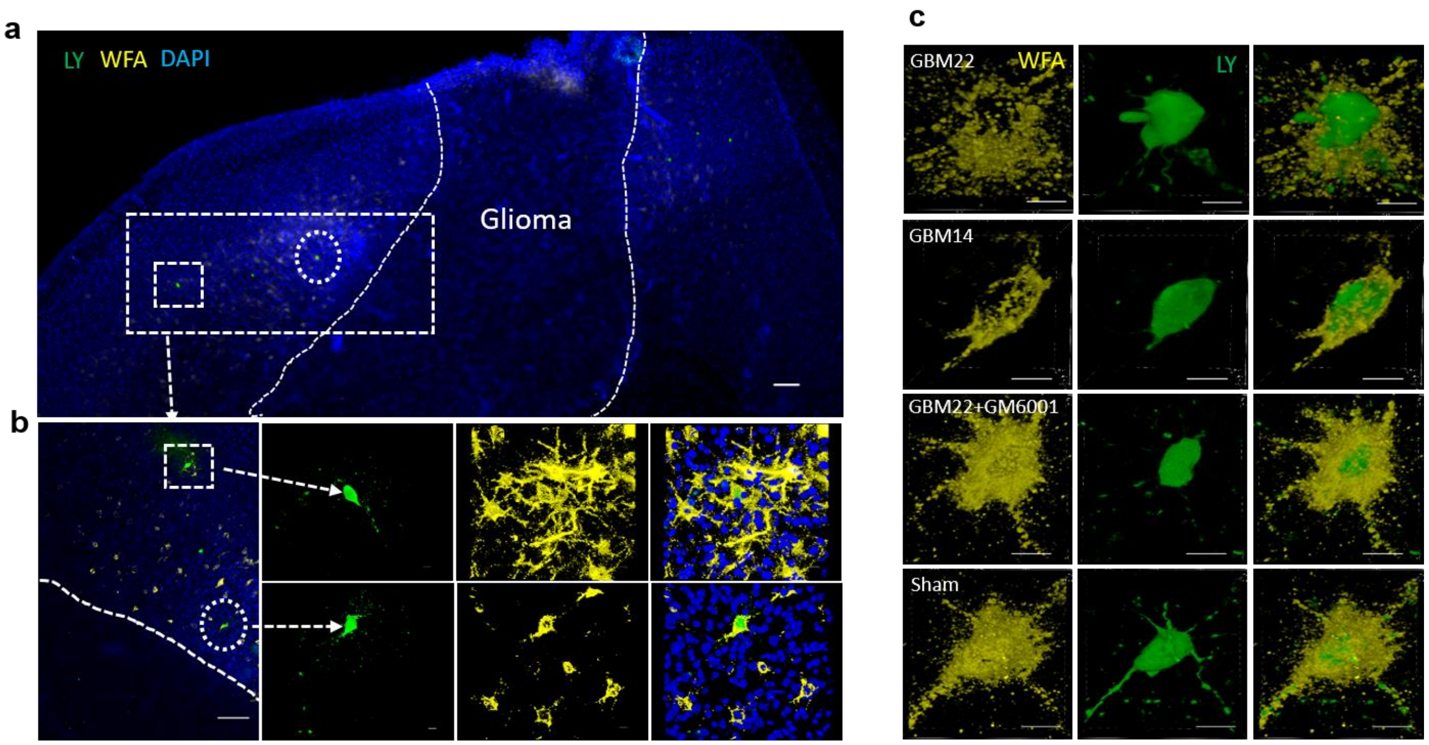

Supplementary Figure 3 Identification of PNN expressing FSNs in peritumoral cortex after electrophysiological recordings. **a** Example photomicrograph of a 300 µm coronal brain slice from a GBM22-implanted animal stained with DAPI (blue) and WFA (yellow) showing a PV^+ peritumoral interneuron that was filled with Lucifer yellow dye (LY) during whole-cell recording. Scale=100 µm. **b** Higher magnification of (a) with WFA staining showing the PV^+ recorded cell (circle) with disintegrated PNN at 131 µm from the tumor border and second recorded PV^+ cell at farther distance from tumor border (477 µm, cell in square) with relatively intact PNN. Scale=100 µm (main image b) and 10 µm in associated panels. **c** 3D volume projection images of recorded and LY-filled PNN expressing FSNs from GBM22 PTC (top), GBM14 PTC (2nd from top), GM6001-treated GBM22 PTC (3rd from top), and sham cortex (bottom) during electrophysiological recordings. Disintegrated PNNs in the peritumoral cortex were within ~200 µm from the tumour border. Scale=5 µm.

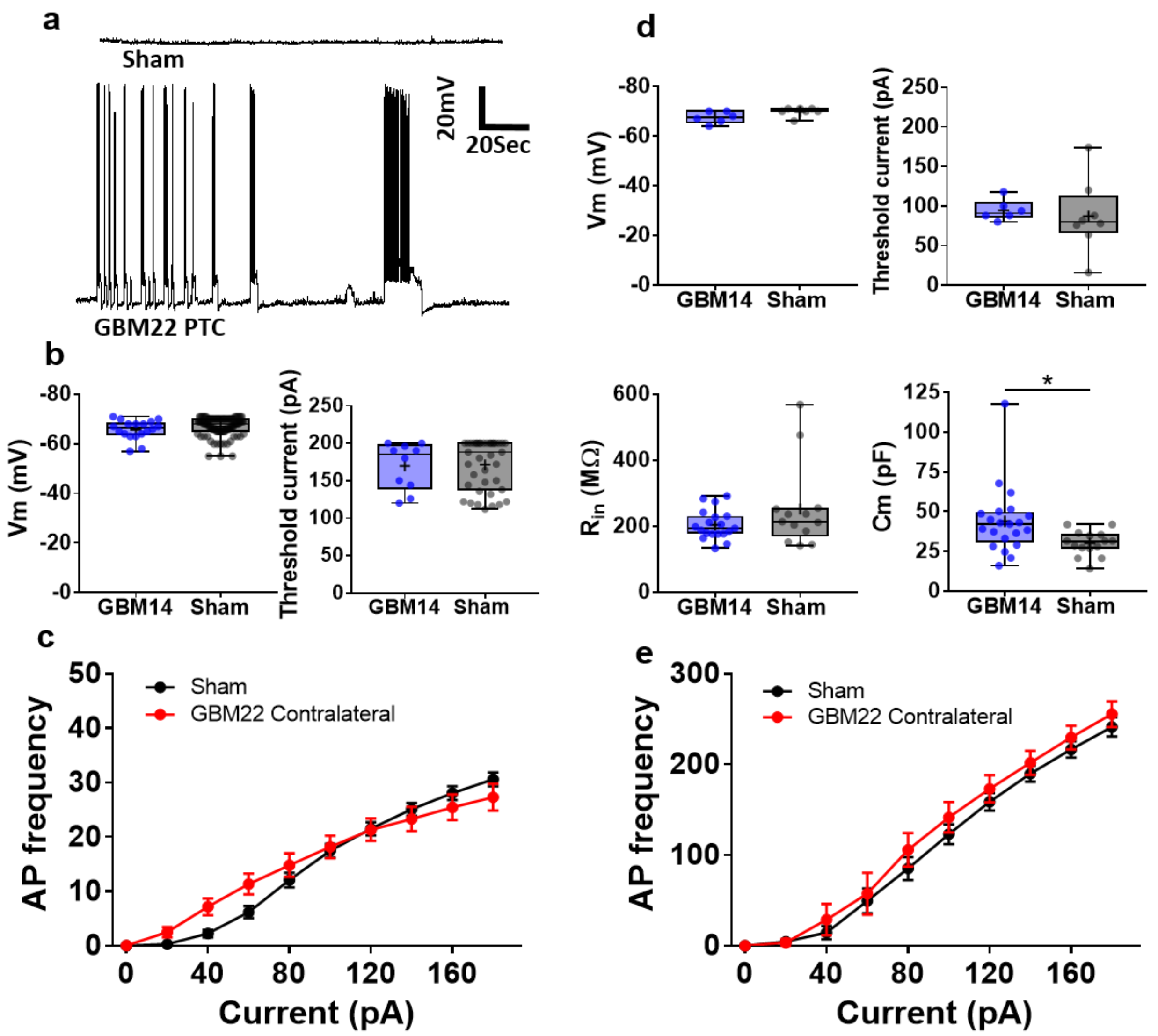

Supplementary Figure 4 Biophysical properties of excitatory neurons and FSNs. **a** Representative current clamp traces of excitatory neuron from sham (top) showing normal resting membrane potential and in GBM22 PTC (Bottom) showing spontaneous epileptiform discharges. **b** Excitatory neurons in GBM14 PTC did not show any significant difference in (left) membrane potential (GBM14, 65.5±1.56, n=10(6)); Sham 67.02±0.38, n=98 (15)), unpaired t test) and (right) threshold current (GBM14, 169.6±9.94, n=10(6)); Sham 171.33±5.14, n=39(10)); unpaired t test). **c** AP frequency response on different depolarization current injections did not show significant difference in GBM22 contralateral excitatory neurons (contralateral n=22(7)); sham n=36(10)); Two-way ANOVA, Sidak's post-hoc test). **d** FSNs in GBM14 PTC exhibited non-significant differences in, (upper left) membrane potential (GBM14, 67.5±0.96, n=6(5); Sham 69.86±0.67, n=7(6); unpaired t test), (upper right) threshold current (GBM14, 94.67±5.41, n=6(5); Sham 87.25±16.07, n=8(5); Welch's t test), and (lower left) input resistance (GBM14, 204.96±10.15, n=19(8); Sham 252.60±35.30, n=13(6); Welch's t test). Membrane capacitance (lower right) (GBM14, 43.89±4.60, n=21(8); Sham 30.11±1.99, n=15(6)) was significantly higher in GBM14 PTC. *P<0.05, Welch's t test. **e** AP frequency response on different depolarization current injection steps was not significant difference in contralateral FSNs (contralateral n=7(5); sham n=10(7); Two-way ANOVA, Sidak's post-hoc test). n=cells(mice) in all. The units for membrane potential (Vm), threshold current, membrane capacitance (Cm) and input resistance (R_{in}) are mV, pA, pF, and MΩ, respectively.

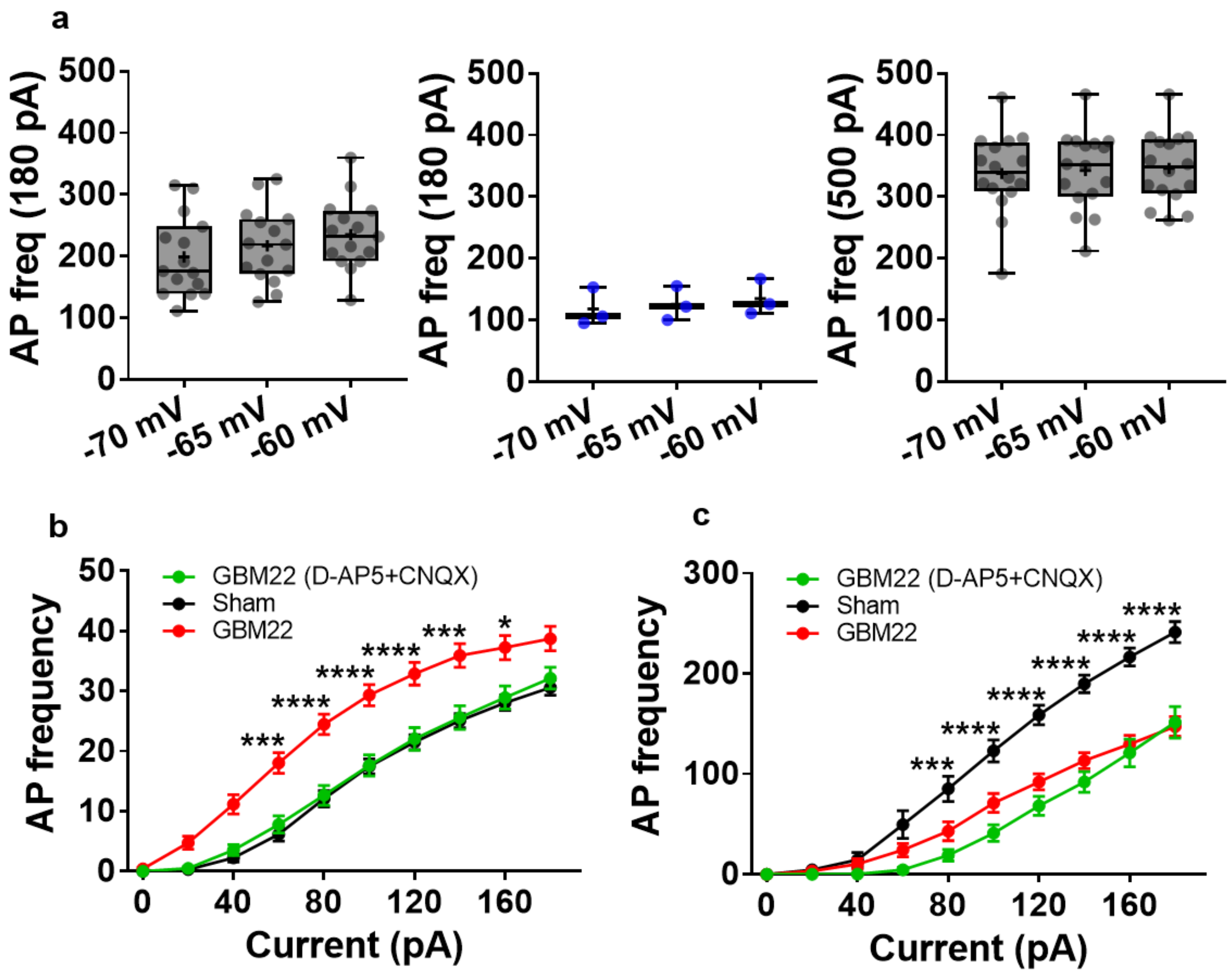

Supplementary Figure 5 Spike firing properties of excitatory and FSNs under different conditions. **a** Action potential frequency on moderate depolarization current injection (180 pA) was not significantly different at membrane holding voltages -70 mV, -65 mV and -60 mV in, (left) shams (-70 mV, 198.90±16.65; -65 mV, 216.70±15.67; -60 mV, 235.10±14.77, n=15(6), One-way ANOVA, Tukey's post-hoc test) and in (middle) GBM22 peritumoral PNN expressing inhibitory neurons (-70 mV, 118±17.79, -65 mV, 125.70±15.98; -60 mV, 134.70±16.74, each voltage n=3(3), One-way ANOVA, Tukey's post-hoc test) suggesting that the decreased spike frequency of inhibitory neurons in GBM22 peritumoral cortex could not be due to depolarized membrane potential. The maximum spike frequency (right) supported by PNN expressing FSNs in sham on higher current injections (500 pA) was also not significantly different at different membrane holding voltages (-70 mV, 337.90±16.24; -65 mV, 342.50±15.89; -60mV, 346.20±14.20, n=16(6), One-way ANOVA, Tukey's post-hoc test). **b** AP frequency of excitatory neurons in GBM22 PTC was reduced significantly in the presence of D-AP5 (50 µM) and CNQX (20 µM). n=27(10) in GBM22, n=27(6) in GBM22 (D-AP5+CNQX), and n=36(10) in sham; Two-way ANOVA, Tukey's post-hoc test. Asterisk (*) shows significant differences in AP frequency between GBM22 and GBM22 (D-AP5+CNQX). **c** AP frequency of FSNs in GBM22 PTC remained unaltered in the presence of APV (50 µM) and CNQX (20 µM). n=18(11) in GBM22, n=17(5) in GBM22 (D-AP5+CNQX), and n=10 (7) in sham; Two-way ANOVA, Tukey's post-hoc test. Asterisk (*) shows significant differences in AP frequency between Sham and GBM22 (D-AP5+CNQX), whereas no differences were found between GBM22 and GBM22 (D-AP5+CNQX). n=cells(mice) in all. AP frequency represents the number of action potentials.

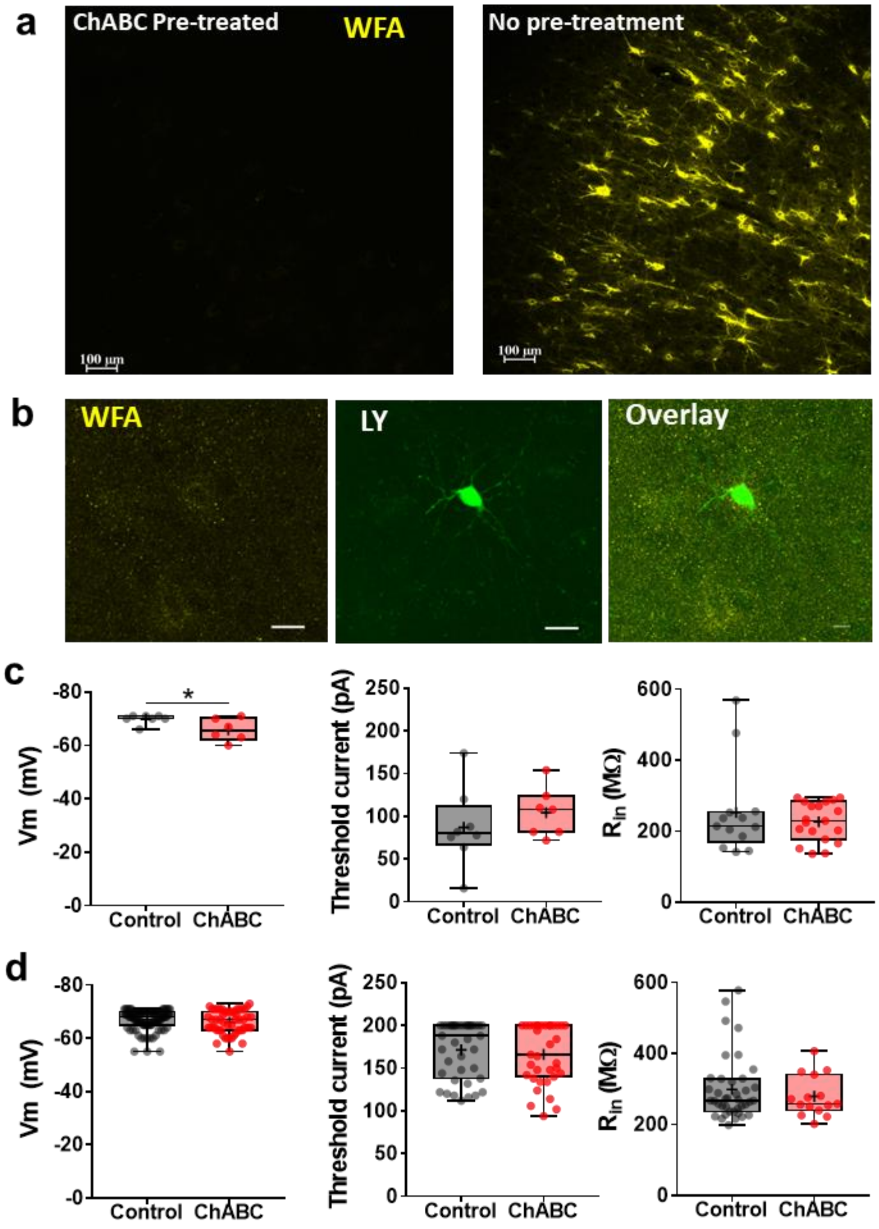

Supplementary Figure 6 ChABC-mediated PNN degradation and effects on FNSs properties in acute cortical slices. **a** Representative WFA (Yellow) staining images of post-recording fixed acute cortical slices, (left) pre-treated with ChABC (0.5 U/ml) showing no WFA-stained PNNs indicating digestion of PNN's CSPGs by ChABC compared to the ACSF control (right) with intact PNNs. Scale=100 µm. **b** Images showing a PNN expressing FSN filled with Lucifer yellow dye (middle) during electrophysiological recording in a ChABC pre-treated slice. The faint outline of WFA (left) around the cell (right) confirms the existence of PNN before ChABC treatment. Scale=20 µm. **c** PNN expressing FSNs in ChABC pre-treated slices exhibited significantly different (left) resting membrane potential (Control 69.86±0.67, n=7(6); ChABC-treated 65.83±1.74, n=6(4); unpaired t test) but unaltered (middle) threshold current (Control 87.25±16.07, n=8(5); ChABC-treated 104.60±10.85, n=7(4); unpaired t test) and (right) input resistance (Control 252.60±35.30, n=13(7); ChABC-treated 226.78±12.58, n=19(7), Welch's t test)**.** **d** Excitatory neurons in the ChABC pre-treated slices showed unaltered (left) resting membrane potential (Control 67.02±0.38, n=98(15); ChABC-treated 66.02±0.58, n=55(8); unpaired t test)**,** (middle) threshold current (Control 171.33±5.28, n=39(15); ChABC-treated 166.11±5.83pA, n=35(7); unpaired t test), and (right) input resistance (Control 298.63±14.47, n=39(15); ChABC-treated 279.13±14.86, n=15(7); unpaired t test). n=cells(mice) in all. The units for membrane potential (Vm), threshold current, and input resistance (R_{in}) are mV, pA, and MΩ, respectively.

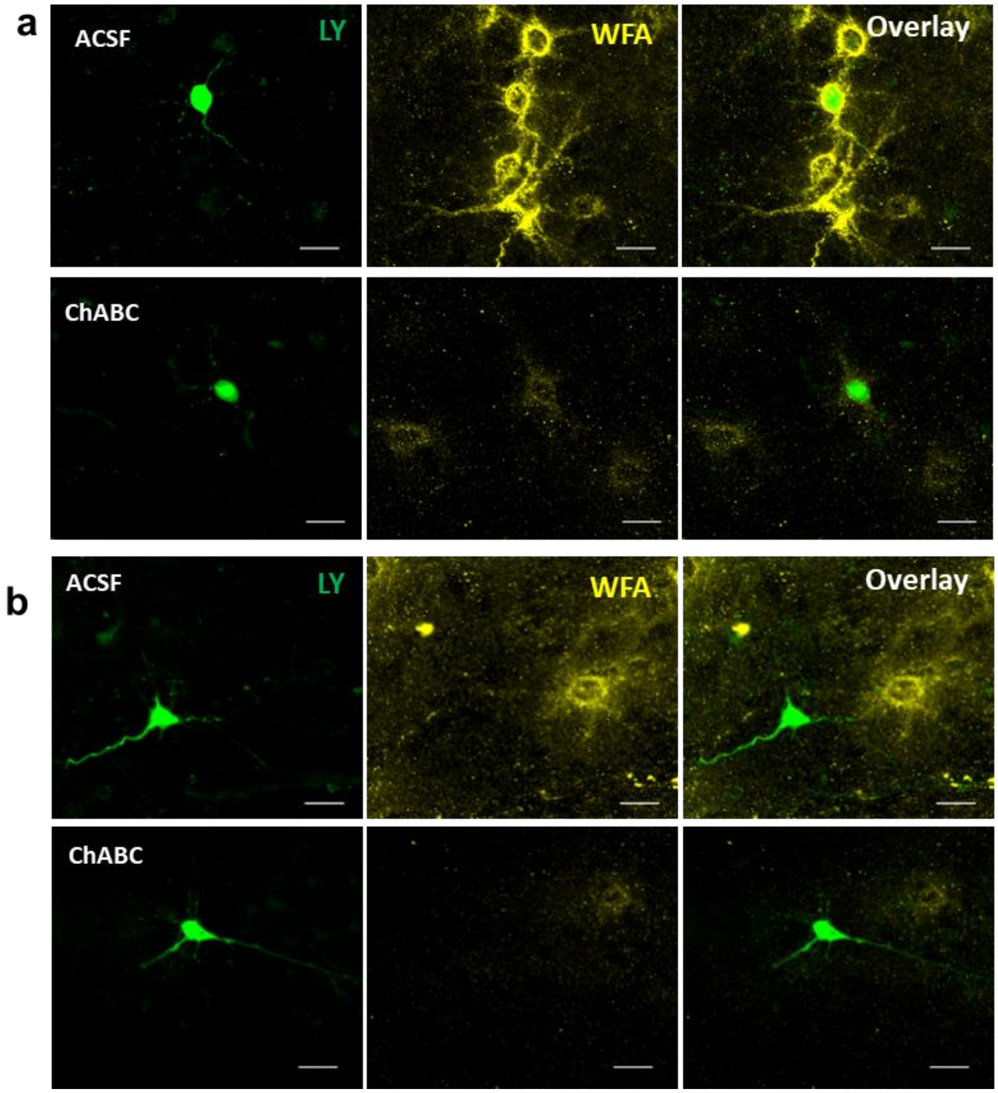

Supplementary Figure 7 Identification of PNN expressing FSNs in ChABC-superfused cortical slices. **a** Patched and recorded FSNs filled with LY (green) showing WFA staining (yellow) around them in ACSF (upper panels) and ChABC (lower panels) superfused cortical slices. Scale=20 µm**. b** Patched and recorded excitatory neurons filled with LY (green) and nearby PNNs (yellow WFA) in ACSF (top panels) and ChABC superfused (bottom panels) cortical slices. Scale=20 µm**.**

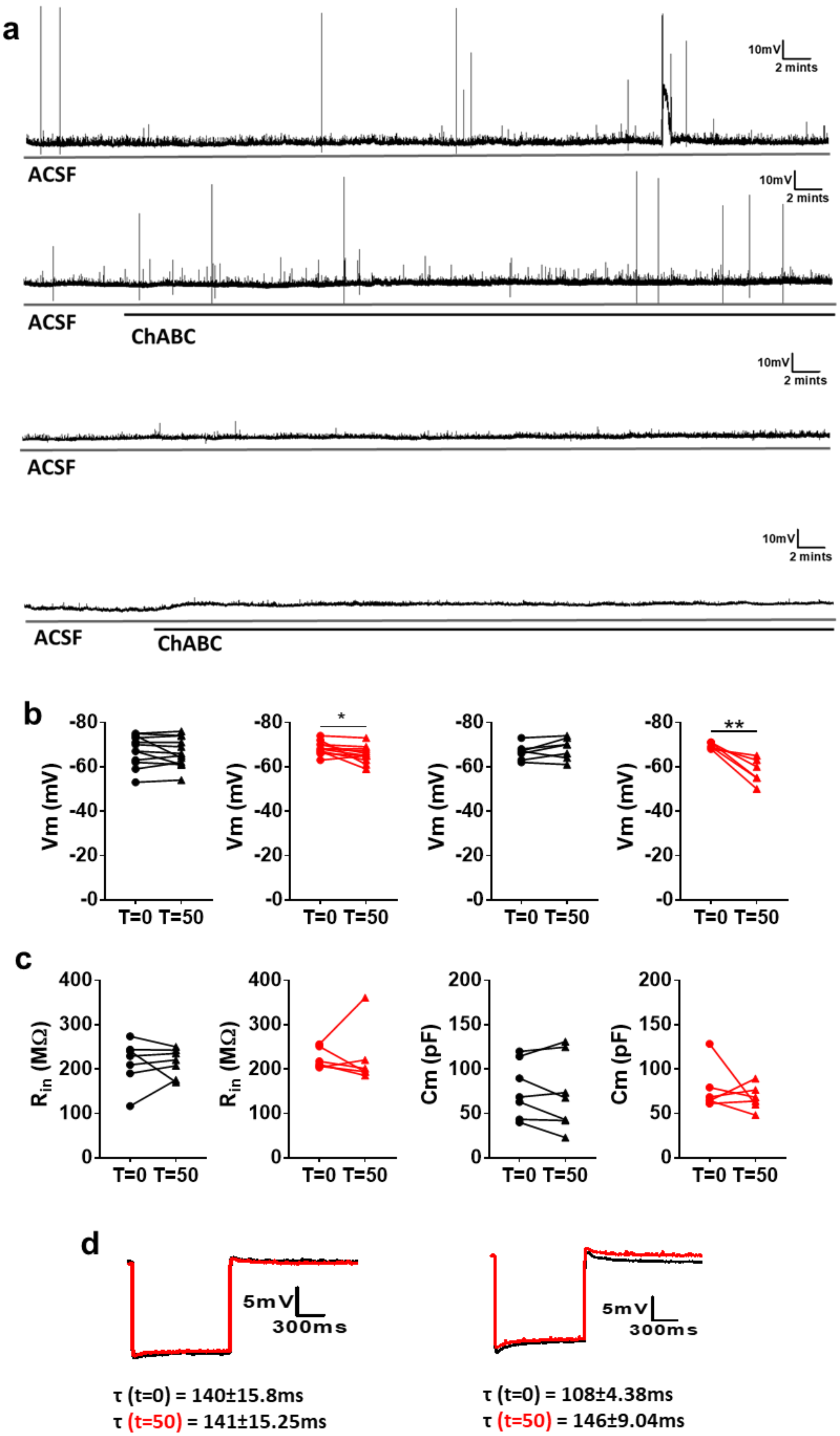
a
10mV
2 mints
ACSF
ChABC
b
Vm (mV)
-80
-60
-40
-20
-0
T=0 T=50
*
**
c
R_{in} (MΩ)
400
300
200
100
0
Cm (pF)
200
150
100
50
d
5mV
300ms
τ (t=0) = 140±15.8ms
τ (t=50) = 141±15.25ms
τ (t=0) = 108±4.38ms
τ (t=50) = 146±9.04ms

Supplementary Figure 8 Properties of PNN expressing FSNs and excitatory neurons during real time ChABC treatment. **a** Representative current clamp recordings of membrane potentials in FSNs in the presence of ACSF (top)**,** and in response to ChABC (2nd from top) superfusion in the cortex. Recordings of excitatory neurons in (3rd from top) ACSF with no change in membrane voltage and (bottom) after ChABC superfusion showing a gradual depolarization of the membrane potential. **b** Summary data showing membrane potential of FSNs in (left) ACSF (T=0 ACSF, 67.42±2.01; T=50 ACSF, 66.42±1.89; n=12(7)) and (2nd from left) after ChABC superfusion (Pre-ChABC, 68.45±0.89; Post-ChABC, 65.54±1.16; n=11(7)). *P<0.05, paired t test. Summary data showing membrane potential of excitatory neurons (3rd from left) during ACSF superfusion (T=0 ACSF, 66.57±1.36; T=50 ACSF, 68.28±1.80; n=7(5)) and (right) after ChABC superfusion (T=0 ChABC, 69.33±0.55; T=50 ChABC, 58±2.30; n=6(4)). **P<0.01, paired t test. **c** R_{in} of excitatory neurons in (left) ACSF (T=0 ACSF, 214.72±19.18; T=50 ACSF, 214.55±12; n=7(5)) and (2nd from left) before and after ChABC (T=0 ChABC, 224.07±9.50; T=50 ChABC, 225.96±27.40; n=6(4)) did not change (paired t test). Cm in (3rd from left) ACSF (T=0 ACSF, 76.90±12.13; T=50 ACSF, 72.02±15.77; n=7(5)) and (right) before and after ChABC treatment (T=0 ChABC 77.62±10.46; T=50 ChABC 67.60±5.77; n=6(4)) also remained unaltered (paired t test). **d** Membrane voltage traces evoked by a hyperpolarizing (-100 pA) current during (left) ACSF (T=0 ACSF (black), T=50 ACSF (red)) and (right) after ChABC superfusion (T=0 ChABC (black), T=50 ChABC (red)) showing unaltered membrane resistance in both but increased membrane time constant (τ) during ChABC superfusion. n=cells(mice) in all. The units for membrane potential (Vm), capacitance (Cm), and input resistance (R_{in}) are mV, pF, and MΩ, respectively.

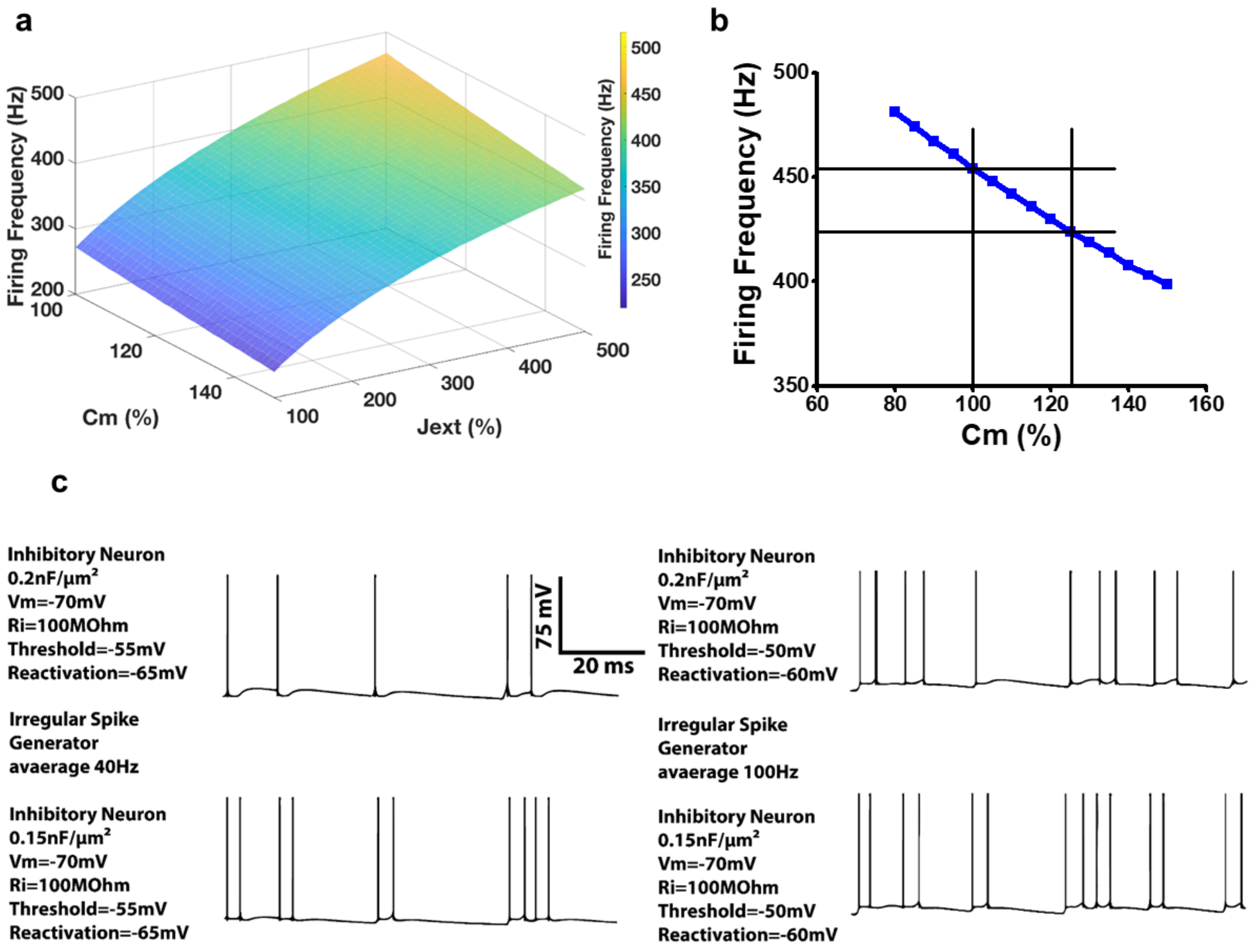

Supplementary Figure 9 Dependence of spike frequency on the membrane capacitance. **a** Matlab generated maximum firing frequency (z-axis) as a function of specific membrane capacitance (Cm, x-axis) and external current (Jext, y-axis) using a Hodgkin Huxley model. (Cm: 100%=1 $\mu F/cm^2$; Jext: 100%=2^{-5} A/cm^2). **b** Matlab generated firing frequency (Hz, y-axis) as a function of specific membrane capacitance at 500% external current injection. Lines bracket the firing frequency for the experimentally observed change in membrane capacitance before and after PNN digestion. **c** Computer simulation of spike output from two inhibitory neurons using Neuronify. Both neurons had identical electrical parameters except those in the bottom traces have a 25% lower specific membrane capacitance. Both were simultaneously recorded and stimulated by an irregular spike generator at 40 Hz (Left) or 100 Hz (right) yielding an average 50% (left bottom) and 30% (right bottom) increase in spike frequency with a 25% decrease in membrane capacitance.

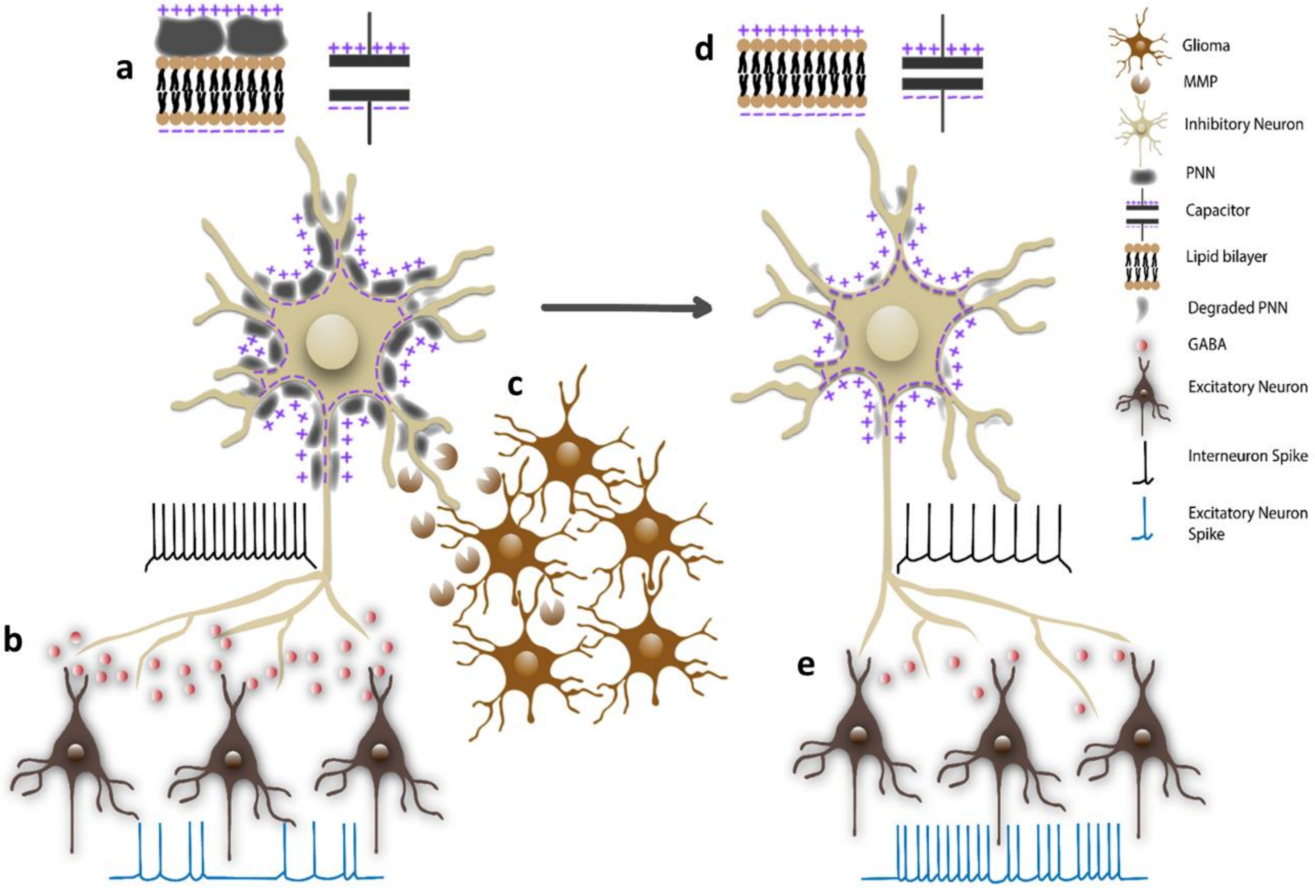

Supplementary Figure 10 Perineuronal nets aid to the membrane capacitance of fast spiking inhibitory neurons to modulate inhibitory tone and subsequently the excitatory output. **a** PNN increases the distance between the charges on the outside and inside the membrane thereby decreasing the specific membrane capacitance. **b** Decreased specific membrane capacitance allows the fast spiking inhibitory neuron to sustain a higher spike frequency resulting in elevated GABA release onto pyramidal excitatory neurons that keeps the overall excitatory drive at bay. **c** Glioma cells release proteases (MMPs) which cleave the PNNs. **d** Cleaving PNN reduces the charge separation distance and increases specific membrane capacitance. **e** Fast spiking inhibitory neurons with degraded PNN have reduced spike frequency causing reduced GABA release leading to excitation inhibition imbalance, which may eventually translate into epileptiform activity.

Supplementary Table 1 Summary of the statistical tests.

Figure	Statistical Test			Multiple comparisons test			Significant?	Summary	95% Confidence interval of difference between group means			Homoscedastic?	Normal distribution?
	Test	Test statistic	p value	Test	Comparisons	Adjusted p value			Mean	Upper limit	Lower limit	(Brown-Forsythe test)	(D'Agostino & Pearson test)
Figure 1b	One-way ANOVA	F = 39.64	<0.0001	Tukey's	0-0.2 mm vs. 0.2-0.4 mm	0.0034	Yes	**	-6.00	-1.54	-10.45	Yes	Yes (except 0.2-0.4 mm)
					0-0.2 mm vs. 0.4-0.6 mm	<0.0001	Yes	****	-9.81	-4.80	-14.81		
					0-0.2 mm vs. Contra	<0.0001	Yes	****	-16.16	-11.59	-20.72		
					0-0.2 mm vs. Sham	<0.0001	Yes	****	-16.86	-12.41	-21.32		
					0.2-0.4 mm vs. 0.4-0.6 mm	0.2272	No	ns	-3.81	1.27	-8.90		
					0.2-0.4 mm vs. Contra	<0.0001	Yes	****	-10.16	-5.51	-14.81		
					0.2-0.4 mm vs. Sham	<0.0001	Yes	****	-10.87	-6.32	-15.41		
					0.4-0.6 mm vs. Contra	0.0091	Yes	**	-6.35	-1.17	-11.52		
					0.4-0.6 mm vs. Sham	0.0023	Yes	**	-7.06	-1.97	-12.14		
					Contra vs. Sham	0.9926	No	ns	-0.71	3.94	-5.36		
Figure 1c	One-way ANOVA	F = 31	<0.0001	Tukey's	0-0.2 mm vs. 0.2-0.4 mm	0.9223	No	ns	-0.20	0.48	-0.87	Yes	Yes
					0-0.2 mm vs. 0.4-0.6 mm	0.1175	No	ns	-0.62	0.09	-1.34		
					0-0.2 mm vs. Contra	<0.0001	Yes	****	-1.94	-1.24	-2.64		
					0-0.2 mm vs. Sham	<0.0001	Yes	****	-2.02	-1.32	-2.72		
					0.2-0.4 mm vs. 0.4-0.6 mm	0.4146	No	ns	-0.43	0.26	-1.12		
					0.2-0.4 mm vs. Contra	<0.0001	Yes	****	-1.75	-1.07	-2.42		
					0.2-0.4 mm vs. Sham	<0.0001	Yes	****	-1.83	-1.15	-2.50		
					0.4-0.6 mm vs. Contra	<0.0001	Yes	****	-1.32	-0.60	-2.04		
					0.4-0.6 mm vs. Sham	<0.0001	Yes	****	-1.40	-0.68	-2.12		
					Contra vs. Sham	0.9975	No	ns	-0.08	0.62	-0.78		
Figure 1d	One-way ANOVA	F = 45.39	<0.0001	Tukey's	0-0.2 mm vs. 0.2-0.4 mm	>0.9999	No	ns	0.00	0.52	-0.53	Yes	Yes
					0-0.2 mm vs. 0.4-0.6 mm	0.0207	Yes	*	-0.61	-0.07	-1.16		
					0-0.2 mm vs. Contra	<0.0001	Yes	****	-1.88	-1.34	-2.41		
					0-0.2 mm vs. Sham	<0.0001	Yes	****	-1.91	-1.31	-2.50		
					0.2-0.4 mm vs. 0.4-0.6 mm	0.0214	Yes	*	-0.61	-0.06	-1.16		
					0.2-0.4 mm vs. Contra	<0.0001	Yes	****	-1.88	-1.34	-2.41		
					0.2-0.4 mm vs. Sham	<0.0001	Yes	****	-1.91	-1.31	-2.50		
					0.4-0.6 mm vs. Contra	<0.0001	Yes	****	-1.26	-0.71	-1.82		
					0.4-0.6 mm vs. Sham	<0.0001	Yes	****	-1.29	-0.68	-1.91		
					Contra vs. Sham	>0.9999	No	ns	-0.03	0.57	-0.64		
Figure 1e	Two-way ANOVA	F = 8.014	0.0005	Tukey's	0-0.2 mm								
		(marker factor)			NeuN vs. PV	0.1964	No	ns	13.22	31.26	-4.83	Yes	Yes (except 0.2-0.4 mm for NeuN+)
					NeuN vs. WFA	0.8321	No	ns	4.15	21.12	-12.82		
					PV vs. WFA	0.4497	No	ns	-9.07	8.68	-26.82		
					0.2-0.4 mm								
					NeuN vs. PV	0.0005	Yes	***	28.64	46.28	11.00		
					NeuN vs. WFA	0.0009	Yes	***	27.02	44.35	9.68		
					PV vs. WFA	0.9723	No	ns	-1.62	15.35	-18.59		
					0.4-0.6 mm								
					NeuN vs. PV	0.0079	Yes	**	26.81	47.70	5.91		
					NeuN vs. WFA	0.0581	No	ns	19.59	39.70	-0.52		
					PV vs. WFA	0.6377	No	ns	-7.22	11.65	-26.08		
					Contra								
					NeuN vs. PV	0.9988	No	ns	0.38	19.16	-18.41		
					NeuN vs. WFA	0.9759	No	ns	-1.61	16.44	-19.66		
					PV vs. WFA	0.9635	No	ns	-1.98	16.07	-20.03		
					Sham								
					NeuN vs. PV	>0.9999	No	ns	0.00	18.39	-18.39		
					NeuN vs. WFA	>0.9999	No	ns	0.00	19.43	-19.43		
					PV vs. WFA	>0.9999	No	ns	0.00	19.80	-19.80		
Figure 2b	One-way ANOVA	F = 34.6	<0.0001	Tukey's	0-0.2 mm vs. 0.2-0.4 mm	0.9048	No	ns	-176.90	392.88	-746.68	No	Yes (except Contra)
					0-0.2 mm vs. 0.4-0.6 mm	<0.0001	Yes	****	-1641.28	-1093.07	-2189.49		
					0-0.2 mm vs. Contra	<0.0001	Yes	****	-1502.22	-919.13	-2085.32		
					0-0.2 mm vs. Sham	<0.0001	Yes	****	-1715.31	-1132.22	-2298.40		
					0.2-0.4 mm vs. 0.4-0.6 mm	<0.0001	Yes	****	-1464.38	-904.46	-2024.31		
					0.2-0.4 mm vs. Contra	<0.0001	Yes	****	-1325.33	-731.21	-1919.45		
					0.2-0.4 mm vs. Sham	<0.0001	Yes	****	-1538.42	-944.29	-2132.54		
					0.4-0.6 mm vs. Contra	0.9592	No	ns	139.06	712.52	-434.41		
					0.4-0.6 mm vs. Sham	0.9961	No	ns	-74.03	499.44	-647.50		
					Contra vs. Sham	0.8589	No	ns	-213.09	393.81	-819.99		
Figure 2e	Unpaired t test	t = 6.792	0.0011	-	-	-	Yes	**	-11.13	-15.34	-6.92	Yes (F test)	n too small
	(two-tailed)												
Figure 3b	Two-way ANOVA	F = 15.12	0.0002	Tukey's	0-0.2 mm:GBM22 vs. 0-0.2 mm:GBM14	>0.9999	No	ns	0.10	0.89	-0.69	Yes (except GBM14)	Yes
		(cell line factor)			0-0.2 mm:GBM22 vs. 0.2-0.4 mm:GBM22	0.9909	No	ns	-0.21	0.58	-1.00		n too small (0.4-0.6 mm for GBM14)
		F = 34.83	<0.0001		0-0.2 mm:GBM22 vs. 0.4-0.6 mm:GBM22	0.5693	No	ns	-0.50	0.33	-1.32		
		(distance factor)			0-0.2 mm:GBM22 vs. Contra:GBM22	<0.0001	Yes	****	-1.84	-1.06	-2.62		
					0-0.2 mm:GBM14 vs. 0.2-0.4 mm:GBM14	<0.0001	Yes	****	-1.62	-0.75	-2.49		
					0-0.2 mm:GBM14 vs. 0.4-0.6 mm:GBM14	<0.0001	Yes	****	-1.78	-0.83	-2.73		
					0-0.2 mm:GBM14 vs. Contra:GBM14	<0.0001	Yes	****	-1.66	-0.92	-2.39		
					0.2-0.4 mm:GBM22 vs. 0.2-0.4 mm:GBM14	0.0003	Yes	***	-1.31	-0.44	-2.18		
					0.2-0.4 mm:GBM22 vs. 0.4-0.6 mm:GBM22	0.9492	No	ns	-0.29	0.51	-1.08		
					0.2-0.4 mm:GBM22 vs. Contra:GBM22	<0.0001	Yes	****	-1.63	-0.88	-2.37		

					0.2-0.4 mm:GBM14 vs. 0.4-0.6 mm:GBM14	0.9998	No	ns	-0.16	0.89	-1.20		
					0.2-0.4 mm:GBM14 vs. Contra:GBM14	>0.9999	No	ns	-0.03	0.81	-0.88		
					0.4-0.6 mm:GBM22 vs. 0.4-0.6 mm:GBM14	0.0076	Yes	**	-1.18	-0.20	-2.16		
					0.4-0.6 mm:GBM22 vs. Contra:GBM22	<0.0001	Yes	****	-1.34	-0.56	-2.12		
					0.4-0.6 mm:GBM14 vs. Contra:GBM14	0.9999	No	ns	0.12	1.06	-0.81		
					Contra:GBM22 vs. Contra:GBM14	0.9255	No	ns	0.28	1.00	-0.44		
Figure 3c	Two-way ANOVA	F = 11.08	0.0013	Tukey's	0-0.2 mm:GBM22 vs. 0-0.2 mm:GBM14	>0.9999	No	ns	-0.06	0.76	-0.87	Yes	Yes
		(cell line factor)			0-0.2 mm:GBM22 vs. 0.2-0.4 mm:GBM22	>0.9999	No	ns	-0.05	0.79	-0.88		n too small (0.4-0.6 mm for GBM14)
		F = 23.7	<0.0001		0-0.2 mm:GBM22 vs. 0.4-0.6 mm:GBM22	0.5033	No	ns	-0.54	0.31	-1.39		
		(distance factor)			0-0.2 mm:GBM22 vs. Contra:GBM22	<0.0001	Yes	****	-1.67	-0.84	-2.50		
					0-0.2 mm:GBM14 vs. 0.2-0.4 mm:GBM14	0.0032	Yes	**	-1.24	-0.28	-2.20		
					0-0.2 mm:GBM14 vs. 0.4-0.6 mm:GBM14	0.0052	Yes	**	-1.41	-0.27	-2.55		
					0-0.2 mm:GBM14 vs. Contra:GBM14	<0.0001	Yes	****	-1.43	-0.63	-2.24		
					0.2-0.4 mm:GBM22 vs. 0.2-0.4 mm:GBM14	0.0034	Yes	**	-1.25	-0.27	-2.23		
					0.2-0.4 mm:GBM22 vs. 0.4-0.6 mm:GBM22	0.6144	No	ns	-0.49	0.35	-1.34		
					0.2-0.4 mm:GBM22 vs. Contra:GBM22	<0.0001	Yes	****	-1.63	-0.79	-2.46		
					0.2-0.4 mm:GBM14 vs. 0.4-0.6 mm:GBM14	0.9999	No	ns	-0.17	1.09	-1.43		
					0.2-0.4 mm:GBM14 vs. Contra:GBM14	0.9986	No	ns	-0.19	0.77	-1.16		
					0.4-0.6 mm:GBM22 vs. 0.4-0.6 mm:GBM14	0.2184	No	ns	-0.93	0.23	-2.08		
					0.4-0.6 mm:GBM22 vs. Contra:GBM22	0.0019	Yes	**	-1.13	-0.28	-1.98		
					0.4-0.6 mm:GBM14 vs. Contra:GBM14	>0.9999	No	ns	-0.02	1.12	-1.16		
					Contra:GBM22 vs. Contra:GBM14	0.9967	No	ns	0.19	1.00	-0.63		
Figure 3e	Welch's t test	t = 7.147	<0.0001	-	-	-	Yes	****	1251	883.70	1619.00	No	Yes
	(two-tailed)												
Figure 3f	Welch's t test	t = 8.411	<0.0001	-	-	-	Yes	****	1377	1018.00	1735.00	No	Yes
	(two-tailed)												
Figure 4b	One-way ANOVA	F = 172.5	<0.0001	Tukey's	<0.3mm PTC vs. >0.6mm PTC	0.2509	No	ns	96.50	242.62	-49.63	No	Yes
					<0.3mm PTC vs. Sham	<0.0001	Yes	****	1029.53	1178.94	880.12		
					>0.6mm PTC vs. Sham	<0.0001	Yes	****	933.03	1082.44	783.62		
Figure 4c	One-way ANOVA	F = 64.54	<0.0001	Tukey's	<0.3mm PTC vs. >0.6mm PTC	<0.0001	Yes	****	-551.71	-403.45	-699.98	Yes	Yes
					<0.3mm PTC vs. Sham	<0.0001	Yes	****	-643.93	-488.62	-799.25		
					>0.6mm PTC vs. Sham	0.3484	No	ns	-92.22	68.07	-252.51		
Figure 4f	Unpaired t test	t = 0.4205	0.6779	-	-	-	No	ns	61.38	-239.90	362.70	Yes (F test)	Yes
	(two-tailed)												
Figure 4g	Welch's t test	t = 7.271	<0.0001	-	-	-	Yes	****	-643.5	-827.30	-459.70	No	Yes
	(two-tailed)												
Figure 5b	One-way ANOVA	F = 38.31	<0.0001	Tukey's	Glioma vs. 0-0.2 mm	<0.0001	Yes	****	802.73	1110.28	495.18	Yes	Yes
					Glioma vs. 0.2-0.4 mm	<0.0001	Yes	****	1002.86	1310.41	695.31		
					Glioma vs. 0.4-0.6 mm	<0.0001	Yes	****	1136.51	1444.06	828.96		
					Glioma vs. Sham	<0.0001	Yes	****	1181.51	1495.67	867.35		
					0-0.2 mm vs. 0.2-0.4 mm	0.3411	No	ns	200.13	500.92	-100.66		
					0-0.2 mm vs. 0.4-0.6 mm	0.0226	Yes	*	333.78	634.57	32.99		
					0-0.2 mm vs. Sham	0.0086	Yes	**	378.78	686.33	71.23		
					0.2-0.4 mm vs. 0.4-0.6 mm	0.7195	No	ns	133.65	434.44	-167.14		
					0.2-0.4 mm vs. Sham	0.479	No	ns	178.65	486.20	-128.90		
					0.4-0.6 mm vs. Sham	0.9937	No	ns	45.00	352.55	-262.55		
Figure 5c	One-way ANOVA	F = 38.2	<0.0001	Tukey's	0-0.2 mm vs. 0.2-0.4 mm	0.122	No	ns	-226.53	42.27	-495.32	Yes	n too small
					0-0.2 mm vs. 0.4-0.6 mm	0.0006	Yes	***	-476.42	-187.48	-765.37		
					0-0.2 mm vs. Sham	<0.0001	Yes	****	-910.08	-658.97	-1161.19		
					0.2-0.4 mm vs. 0.4-0.6 mm	0.0939	No	ns	-249.90	30.59	-530.39		
					0.2-0.4 mm vs. Sham	<0.0001	Yes	****	-683.56	-442.23	-924.89		
					0.4-0.6 mm vs. Sham	0.0006	Yes	***	-433.66	-170.07	-697.25		
Figure 5d	Two-way ANOVA	F = 47.5	P<0.0001	Sidak's	GBM22 - +GM6001							Yes (excpet +GM6001)	Yes
					Glioma	<0.0001	Yes	****	661.61	911.74	411.48		
					PTC	0.0023	Yes	**	255.87	428.57	83.18		
Figure 5e	Two-way ANOVA	F = 40.06	P<0.0001	Sidak's	GBM22 - +GM6001							Yes (except GBM22)	Yes
					0-0.2 mm	0.0003	Yes	***	-0.71	-0.28	-1.13		
					0.2-0.4 mm	0.0001	Yes	***	-0.75	-0.33	-1.18		
					0.4-0.6 mm	0.0244	Yes	*	-0.52	-0.05	-1.00		
Figure 5h	One-way ANOVA	F = 47.01	<0.0001	Tukey's	GBM22 vs. +GM6001	<0.0001	Yes	****	-8.02	-5.46	-10.58	Yes	n tool small
					GBM22 vs. Sham	<0.0001	Yes	****	-8.96	-6.29	-11.62		
					+GM6001 vs. Sham	0.6432	No	ns	-0.94	1.72	-3.61		
Figure 5i	One-way ANOVA	F = 65.61	<0.0001	Tukey's	GBM22 vs. +GM6001	0.026	Yes	*	-163.50	-16.60	-310.39	Yes	Yes
					GBM22 vs. Sham	<0.0001	Yes	****	-789.27	-615.13	-963.41		
					+GM6001 vs. Sham	<0.0001	Yes	****	-625.77	-463.78	-787.77		
Figure 6a	One-way ANOVA	F = 21.87	<0.0001	Tukey's	PTA vs. Contra	0.0003	Yes	***	-5.51	-2.22	-8.81	No	No
					PTA vs. Sham	<0.0001	Yes	****	-5.53	-3.47	-7.59		
					Contra vs. Sham	0.9999	No	ns	-0.02	3.21	-3.25		
Figure 6b	One-way ANOVA	F = 0.2043	0.8158	Tukey's	PTA vs. Contra	0.8851	No	ns	5.44	33.20	-22.32	Yes	Yes (except PTA and Contra)

						PTA vs. Sham	0.9946	No	ns	-1.03	24.03	-26.09		
						Contra vs. Sham	0.8094	No	ns	-6.47	18.59	-31.52		
Figure 6c	One-way ANOVA	F = 0.5981	0.5526	Tukey's		PTA vs. Contra	0.7953	No	ns	18.96	89.25	-51.33	Yes	Yes (except Sham)
						PTA vs. Sham	0.8733	No	ns	-10.45	39.96	-60.86		
						Contra vs. Sham	0.5322	No	ns	-29.41	36.05	-94.86		
Figure 6d	One-way ANOVA	F = 6.12	0.0036	Tukey's		PTA vs. Contra	0.0054	Yes	**	-42.79	-11.06	-74.52	Yes	Yes (except Sham)
						PTA vs. Sham	0.007	Yes	**	-36.56	-8.65	-64.48		
						Contra vs. Sham	0.8204	No	ns	6.22	31.06	-18.62		
Figure 6e	Two-way ANOVA	F = 105.2	<0.0001	Tukey's	0								Yes (except Sham and GBM22)	Yes (about half of the groups)
						Sham vs. GBM14	>0.9999	No	ns	0.00	5.44	-5.44		
						Sham vs. GBM22	0.9721	No	ns	-0.44	4.16	-5.05		
						GBM14 vs. GBM22	0.9817	No	ns	-0.44	5.26	-6.15		
					20									
						Sham vs. GBM14	0.6614	No	ns	-2.01	3.43	-7.44		
						Sham vs. GBM22	0.0593	No	ns	-4.47	0.13	-9.08		
						GBM14 vs. GBM22	0.5682	No	ns	-2.47	3.24	-8.17		
					40									
						Sham vs. GBM14	0.1347	No	ns	-4.44	1.00	-9.87		
						Sham vs. GBM22	<0.0001	Yes	****	-8.94	-4.33	-13.54		
						GBM14 vs. GBM22	0.1542	No	ns	-4.50	1.21	-10.21		
					60									
						Sham vs. GBM14	0.0308	Yes	*	-5.87	-0.43	-11.31		
						Sham vs. GBM22	<0.0001	Yes	****	-11.88	-7.27	-16.49		
						GBM14 vs. GBM22	0.0363	Yes	*	-6.01	-0.30	-11.72		
					80									
						Sham vs. GBM14	0.145	No	ns	-4.35	1.08	-9.79		
						Sham vs. GBM22	<0.0001	Yes	****	-12.40	-7.79	-17.01		
						GBM14 vs. GBM22	0.0028	Yes	**	-8.04	-2.33	-13.75		
					100									
						Sham vs. GBM14	0.3617	No	ns	-3.15	2.28	-8.59		
						Sham vs. GBM22	<0.0001	Yes	****	-11.86	-7.25	-16.47		
						GBM14 vs. GBM22	0.0011	Yes	**	-8.71	-3.00	-14.42		
					120									
						Sham vs. GBM14	0.4289	No	ns	-2.88	2.56	-8.31		
						Sham vs. GBM22	<0.0001	Yes	****	-11.39	-6.78	-16.00		
						GBM14 vs. GBM22	0.0014	Yes	**	-8.51	-2.80	-14.22		
					140									
						Sham vs. GBM14	0.5495	No	ns	-2.42	3.02	-7.85		
						Sham vs. GBM22	<0.0001	Yes	****	-10.84	-6.24	-15.45		
						GBM14 vs. GBM22	0.0016	Yes	**	-8.43	-2.72	-14.13		
					160									
						Sham vs. GBM14	0.6102	No	ns	-2.19	3.24	-7.63		
						Sham vs. GBM22	<0.0001	Yes	****	-9.20	-4.60	-13.81		
						GBM14 vs. GBM22	0.0113	Yes	*	-7.01	-1.30	-12.72		
					180									
						Sham vs. GBM14	0.7677	No	ns	-1.60	3.83	-7.04		
						Sham vs. GBM22	0.0001	Yes	***	-8.16	-3.55	-12.76		
						GBM14 vs. GBM22	0.0196	Yes	*	-6.55	-0.84	-12.26		
Figure 6g	One-way ANOVA	F = 15.8	<0.0001	Tukey's		PTA vs. Contra	0.0014	Yes	**	-6.48	-2.47	-10.48	Yes	Yes (PTA)
						PTA vs. Sham	<0.0001	Yes	****	-8.19	-4.18	-12.20		n too small (Contra, Sham)
						Contra vs. Sham	0.613	No	ns	-1.71	2.79	-6.22		
Figure 6h	One-way ANOVA	F = 8.69	0.0009	Tukey's		PTA vs. Contra	0.0038	Yes	**	15.03	25.58	4.47	No	Yes
						PTA vs. Sham	0.0025	Yes	**	13.73	22.96	4.51		
						Contra vs. Sham	0.949	No	ns	-1.29	8.97	-11.55		
Figure 6i	One-way ANOVA	F = 0.08183	0.9216	Tukey's		PTA vs. Contra	0.9576	No	ns	9.03	87.56	-69.51	Yes	Yes (except Sham)
						PTA vs. Sham	0.9936	No	ns	-3.53	76.44	-83.51		
						Contra vs. Sham	0.9196	No	ns	-12.56	65.97	-91.09		
Figure 6j	One-way ANOVA	F = 0.4693	0.6322	Tukey's		PTA vs. Contra	0.6225	No	ns	-17.82	30.19	-65.83	Yes	Yes (except Contra)
						PTA vs. Sham	0.9674	No	ns	-4.50	41.88	-50.88		
						Contra vs. Sham	0.7651	No	ns	13.32	61.33	-34.69		
Figure 6k	Two-way ANOVA	F = 104.8	<0.0001	Tukey's	0								Yes (except Sham and GBM22)	Yes (about half of the groups)
						Sham vs. GBM14	>0.9999	No	ns	0.00	28.00	-28.00		n too small (GBM14)
						Sham vs. GBM22	>0.9999	No	ns	-0.11	28.16	-28.38		
						GBM14 vs. GBM22	>0.9999	No	ns	-0.11	23.47	-23.69		
					20									
						Sham vs. GBM14	0.9262	No	ns	4.44	32.45	-23.56		
						Sham vs. GBM22	0.9939	No	ns	1.27	29.54	-27.00		
						GBM14 vs. GBM22	0.9462	No	ns	-3.18	20.40	-26.75		
					40									
						Sham vs. GBM14	0.7423	No	ns	8.76	36.77	-19.24		
						Sham vs. GBM22	0.9392	No	ns	4.06	32.33	-24.22		
						GBM14 vs. GBM22	0.8856	No	ns	-4.71	18.87	-28.28		
					60									
						Sham vs. GBM14	0.0263	Yes	*	30.92	58.92	2.91		
						Sham vs. GBM22	0.0897	No	ns	25.32	53.59	-2.95		

						GBM14 vs. GBM22	0.8425	No	ns	-5.59	17.98	-29.17		
					80									
						Sham vs. GBM14	<0.0001	Yes	****	52.25	80.25	24.24		
						Sham vs. GBM22	0.0014	Yes	**	42.30	70.57	14.03		
						GBM14 vs. GBM22	0.5822	No	ns	-9.95	13.63	-33.52		
					100									
						Sham vs. GBM14	<0.0001	Yes	****	67.68	95.69	39.68		
						Sham vs. GBM22	<0.0001	Yes	****	51.78	80.05	23.51		
						GBM14 vs. GBM22	0.2525	No	ns	-15.91	7.67	-39.48		
					120									
						Sham vs. GBM14	<0.0001	Yes	****	76.69	104.70	48.69		
						Sham vs. GBM22	<0.0001	Yes	****	66.58	94.85	38.31		
						GBM14 vs. GBM22	0.5715	No	ns	-10.12	13.46	-33.69		
					140									
						Sham vs. GBM14	<0.0001	Yes	****	89.48	117.48	61.47		
						Sham vs. GBM22	<0.0001	Yes	****	76.46	104.73	48.18		
						GBM14 vs. GBM22	0.3965	No	ns	-13.02	10.55	-36.60		
					160									
						Sham vs. GBM14	<0.0001	Yes	****	94.38	122.39	66.38		
						Sham vs. GBM22	<0.0001	Yes	****	86.87	115.14	58.60		
						GBM14 vs. GBM22	0.7339	No	ns	-7.52	16.06	-31.09		
					180									
						Sham vs. GBM14	<0.0001	Yes	****	103.93	131.93	75.92		
						Sham vs. GBM22	<0.0001	Yes	****	94.12	122.39	65.85		
						GBM14 vs. GBM22	0.5912	No	ns	-9.80	13.77	-33.38		
Figure 7a	One-way ANOVA	F = 7.307	0.0023	Tukey's		GBM22 vs. +GM6001	0.7686	No	ns	-1.22	3.09	-5.54	Yes	Yes (GBM22); No (+GM6001)
						GBM22 vs. Sham	0.0025	Yes	**	-8.19	-2.68	-13.70		n too small (Sham)
						+GM6001 vs. Sham	0.0061	Yes	**	-6.97	-1.81	-12.13		
Figure 7b	One-way ANOVA	F = 12.67	<0.0001	Tukey's		GBM22 vs. +GM6001	<0.0001	Yes	****	15.56	23.42	7.71	No	Yes
						GBM22 vs. Sham	0.0007	Yes	***	13.73	22.09	5.38		
						+GM6001 vs. Sham	0.8269	No	ns	-1.83	5.71	-9.36		
Figure 7c	One-way ANOVA	F = 0.6978	0.5032	Tukey's		GBM22 vs. +GM6001	0.6267	No	ns	29.21	105.73	-47.31	Yes	Yes (except Sham)
						GBM22 vs. Sham	0.9943	No	ns	-3.53	80.71	-87.78		
						+GM6001 vs. Sham	0.5569	No	ns	-32.74	43.78	-109.26		
Figure 7d	One-way ANOVA	F = 3.501	0.0451	Tukey's		GBM22 vs. +GM6001	0.0697	No	ns	-38.02	2.58	-78.62	Yes	Yes
						GBM22 vs. Sham	0.9668	No	ns	-4.50	40.68	-49.68		
						+GM6001 vs. Sham	0.1201	No	ns	33.52	74.12	-7.08		
Figure 7e	Two-way ANOVA	F = 42.53	<0.0001	Tukey's	0								No	Yes (about half of the groups)
						Sham vs. GBM22	>0.9999	No	ns	-0.11	44.13	-44.36		
						Sham vs. +GM6001	>0.9999	No	ns	0.00	48.03	-48.03		
						GBM22 vs. +GM6001	>0.9999	No	ns	0.11	41.92	-41.70		
					20									
						Sham vs. GBM22	0.9975	No	ns	1.27	45.51	-42.98		
						Sham vs. +GM6001	0.9789	No	ns	4.02	52.05	-44.02		
						GBM22 vs. +GM6001	0.9869	No	ns	2.75	44.56	-39.06		
					40									
						Sham vs. GBM22	0.9747	No	ns	4.06	48.30	-40.19		
						Sham vs. +GM6001	0.9997	No	ns	-0.50	47.53	-48.53		
						GBM22 vs. +GM6001	0.9644	No	ns	-4.56	37.25	-46.36		
					60									
						Sham vs. GBM22	0.3703	No	ns	25.32	69.57	-18.92		
						Sham vs. +GM6001	0.5331	No	ns	21.85	69.88	-26.18		
						GBM22 vs. +GM6001	0.9792	No	ns	-3.47	38.34	-45.28		
					80									
						Sham vs. GBM22	0.0644	No	ns	42.30	86.55	-1.95		
						Sham vs. +GM6001	0.9289	No	ns	7.47	55.50	-40.57		
						GBM22 vs. +GM6001	0.1235	No	ns	-34.83	6.98	-76.64		
					100									
						Sham vs. GBM22	0.017	Yes	*	51.78	96.02	7.53		
						Sham vs. +GM6001	0.9773	No	ns	4.17	52.20	-43.87		
						GBM22 vs. +GM6001	0.021	Yes	*	-47.61	-5.80	-89.42		
					120									
						Sham vs. GBM22	0.0013	Yes	**	66.58	110.82	22.33		
						Sham vs. +GM6001	0.999	No	ns	0.88	48.92	-47.15		
						GBM22 vs. +GM6001	0.0007	Yes	***	-65.69	-23.89	-107.50		
					140									
						Sham vs. GBM22	0.0002	Yes	***	76.46	120.70	32.21		
						Sham vs. +GM6001	0.9867	No	ns	-3.18	44.85	-51.22		
						GBM22 vs. +GM6001	<0.0001	Yes	****	-79.64	-37.83	-121.45		
					160									
						Sham vs. GBM22	<0.0001	Yes	****	86.87	131.11	42.62		
						Sham vs. +GM6001	0.8919	No	ns	-9.30	38.73	-57.33		
						GBM22 vs. +GM6001	<0.0001	Yes	****	-96.17	-54.36	-137.98		
					180									
						Sham vs. GBM22	<0.0001	Yes	****	94.12	138.37	49.88		
						Sham vs. +GM6001	0.804	No	ns	-12.85	35.18	-60.88		
						GBM22 vs. +GM6001	<0.0001	Yes	****	-106.97	-65.16	-148.78		

Figure 7g	One-way ANOVA	F = 22.86	<0.0001	Tukey's		GBM22 vs. +GM6001	0.0002	Yes	***	-5.14	-2.18	-8.10	No	No
						GBM22 vs. Sham	<0.0001	Yes	****	-5.53	-3.53	-7.54		
						+GM6001 vs. Sham	0.9454	No	ns	-0.39	2.50	-3.28		
Figure 7h	One-way ANOVA	F = 0.2721	0.7626	Tukey's		GBM22 vs. +GM6001	0.8597	No	ns	5.89	32.83	-21.04	Yes	Yes (except GBM22 and +GM6001)
						GBM22 vs. Sham	0.9953	No	ns	-1.03	25.71	-27.77		
						+GM6001 vs. Sham	0.7648	No	ns	-6.92	16.81	-30.65		
Figure 7i	One-way ANOVA	F = 4.223	0.0178	Tukey's		GBM22 vs. +GM6001	0.1139	No	ns	43.54	94.98	-7.91	Yes	Yes (except Sham)
						GBM22 vs. Sham	0.8604	No	ns	-10.45	37.21	-58.11		
						+GM6001 vs. Sham	0.0155	Yes	*	-53.99	-8.60	-99.38		
Figure 7j	One-way ANOVA	F = 8.003	0.0007	Tukey's		GBM22 vs. +GM6001	0.948	No	ns	-3.98	26.60	-34.57	Yes	Yes (except Sham)
						GBM22 vs. Sham	0.0082	Yes	**	-36.56	-8.12	-65.01		
						+GM6001 vs. Sham	0.0033	Yes	**	-32.58	-9.54	-55.63		
Figure 7k	Two-way ANOVA	F = 109.3	<0.0001	Tukey's	0								No	Yes (about half of the groups)
						Sham vs. GBM22	0.971	No	ns	-0.44	4.07	-4.96		
						Sham vs. +GM6001	>0.9999	No	ns	0.00	4.62	-4.62		
						GBM22 vs. +GM6001	0.9756	No	ns	0.44	5.37	-4.48		
					20									
						Sham vs. GBM22	0.0531	No	ns	-4.47	0.05	-8.99		
						Sham vs. +GM6001	0.9802	No	ns	-0.37	4.25	-4.99		
						GBM22 vs. +GM6001	0.1246	No	ns	4.10	9.02	-0.83		
					40									
						Sham vs. GBM22	<0.0001	Yes	****	-8.94	-4.42	-13.45		
						Sham vs. +GM6001	0.6728	No	ns	-1.67	2.95	-6.29		
						GBM22 vs. +GM6001	0.0016	Yes	**	7.27	12.19	2.34		
					60									
						Sham vs. GBM22	<0.0001	Yes	****	-11.88	-7.36	-16.40		
						Sham vs. +GM6001	0.1354	No	ns	-3.77	0.85	-8.39		
						GBM22 vs. +GM6001	0.0003	Yes	***	8.11	13.04	3.19		
					80									
						Sham vs. GBM22	<0.0001	Yes	****	-12.40	-7.88	-16.92		
						Sham vs. +GM6001	0.0964	No	ns	-4.08	0.54	-8.70		
						GBM22 vs. +GM6001	0.0002	Yes	***	8.32	13.25	3.40		
					100									
						Sham vs. GBM22	<0.0001	Yes	****	-11.86	-7.34	-16.38		
						Sham vs. +GM6001	0.0391	Yes	*	-4.81	-0.19	-9.43		
						GBM22 vs. +GM6001	0.0023	Yes	**	7.05	11.98	2.13		
					120									
						Sham vs. GBM22	<0.0001	Yes	****	-11.39	-6.87	-15.91		
						Sham vs. +GM6001	0.0017	Yes	**	-6.78	-2.16	-11.40		
						GBM22 vs. +GM6001	0.0723	No	ns	4.61	9.53	-0.32		
					140									
						Sham vs. GBM22	<0.0001	Yes	****	-10.84	-6.32	-15.36		
						Sham vs. +GM6001	0.0005	Yes	***	-7.40	-2.78	-12.02		
						GBM22 vs. +GM6001	0.2284	No	ns	3.45	8.37	-1.48		
					160									
						Sham vs. GBM22	<0.0001	Yes	****	-9.20	-4.69	-13.72		
						Sham vs. +GM6001	<0.0001	Yes	****	-8.34	-3.72	-12.96		
						GBM22 vs. +GM6001	0.9117	No	ns	0.86	5.78	-4.07		
					180									
						Sham vs. GBM22	<0.0001	Yes	****	-8.16	-3.64	-12.68		
						Sham vs. +GM6001	<0.0001	Yes	****	-8.90	-4.28	-13.52		
						GBM22 vs. +GM6001	0.9339	No	ns	-0.74	4.19	-5.66		
Figure 8a	Two-way ANOVA	F = 82.03	<0.0001	Sidak's	Control - Control+ChABC								No	Yes
(AP freq)						0	>0.9999	No	ns	0.00	47.59	-47.59		
						20	>0.9999	No	ns	4.60	52.19	-42.99		
						40	0.9927	No	ns	14.50	62.09	-33.09		
						60	0.0795	No	ns	44.85	92.44	-2.74		
						80	0.0011	Yes	**	66.68	114.27	19.08		
						100	0.0002	Yes	***	72.88	120.47	25.28		
						120	<0.0001	Yes	****	84.68	132.27	37.08		
						140	<0.0001	Yes	****	77.03	124.62	29.43		
						160	0.0046	Yes	**	59.95	107.54	12.36		
						180	0.0126	Yes	*	55.03	102.62	7.43		
Figure 8a	Unpaired t test	t = 2.518	0.0186	-		-	-	Yes	*	9.77	1.78	17.76	Yes (F test)	Yes
(Cm)	(two-tailed)													
Figure 8b	Two-way ANOVA	F = 56.26	<0.0001	Sidak's	Control - Control+ChABC								No	Yes (Control+ChABC)
(AP freq)						0	>0.9999	No	ns	-0.18	5.53	-5.89		Some data set (Control)
						20	>0.9999	No	ns	-0.97	4.74	-6.68		
						40	0.5459	No	ns	-3.61	2.10	-9.33		
						60	0.0498	Yes	*	-5.71	0.00	-11.43		
						80	0.0517	No	ns	-5.69	0.02	-11.40		
						100	0.2337	No	ns	-4.53	1.18	-10.24		
						120	0.0399	Yes	*	-5.86	-0.15	-11.58		
						140	0.0154	Yes	*	-6.46	-0.75	-12.17		
						160	0.0049	Yes	**	-7.13	-1.41	-12.84		
						180	0.0008	Yes	***	-8.05	-2.34	-13.76		

Figure 8b<br>(Cm)	Unpaired t test<br>(two-tailed)	t = 0.5056	0.6165	-	-	-	No	ns	-7.092	-35.63	21.45	Yes (F test)	Yes
Figure 8c<br>(Rin)	Paired t test<br>(two-tailed)	t = 0.5007	0.6301	-	-	-	No	ns	2.781	-10.03	15.59	Effective pairing	Yes
Figure 8c<br>(Cm)	Paired t test<br>(two-tailed)	t = 6.979	0.0001	-	-	-	Yes	***	12.23	8.19	16.27	Effective pairing	Yes
Figure 8c<br>(Max APs)	Paired t test<br>(two-tailed)	t = 3.435	0.0109	-	-	-	Yes	*	-84.13	-142.00	-26.21	No effective pairing	Yes (except T=50)
Figure 8f<br>(Rin)	Paired t test<br>(two-tailed)	t = 0.4881	0.6385	-	-	-	No	ns	2.914	-10.85	16.68	Effective pairing	Yes
Figure 8f<br>(Cm)	Paired t test<br>(two-tailed)	t = 0.4815	0.6431	-	-	-	No	ns	0.59	-2.24	3.43	Effective pairing	Yes
Figure 8f<br>(Max APs)	Paired t test<br>(two-tailed)	t = 1.117	0.3009	-	-	-	No	ns	-3.50	-10.91	3.91	Effective pairing	Yes
Figure 9f	Unpaired t test<br>(two-tailed)	t = 1.705	0.1054	-	-	-	No	ns	-2.46	-5.49	0.57	Yes (F test)	Yes
Figure 9g	Welch's t test<br>(two-tailed)	t = 4.642	0.0003	-	-	-	Yes	***	-34.83	-50.75	-18.90	No	Yes
Supplementary Figure 1c<br>($WFA^{+}NeuN^{+}PV^{-}$)	One-way ANOVA	F = 0.6204	0.6073	Sidak's	0.0-0.2 mm vs. 0.2-0.4 mm	>0.9999	No	ns	-0.01	2.12	-2.14	Yes	No
					0.0-0.2 mm vs. 0.4-0.6 mm	0.9325	No	ns	0.70	2.83	-1.43		
					0.0-0.2 mm vs. Sham	0.9098	No	ns	0.80	3.07	-1.47		
					0.2-0.4 mm vs. 0.4-0.6 mm	0.9281	No	ns	0.71	2.84	-1.42		
					0.2-0.4 mm vs. Sham	0.9049	No	ns	0.81	3.08	-1.46		
					0.4-0.6 mm vs. Sham	>0.9999	No	ns	0.10	2.37	-2.17		
Supplementary Figure 1c<br>(PV^{+}/WFA^{+})	One-way ANOVA	F = 0.5969	0.6221	Sidak's	0.0-0.2 mm vs. 0.2-0.4 mm	0.8777	No	ns	0.23	0.83	-0.38	Yes	Yes (except 0.0-0.2 mm)
					0.0-0.2 mm vs. 0.4-0.6 mm	>0.9999	No	ns	-0.03	0.58	-0.63		
					0.0-0.2 mm vs. Sham	>0.9999	No	ns	0.05	0.70	-0.59		
					0.2-0.4 mm vs. 0.4-0.6 mm	0.7954	No	ns	-0.25	0.33	-0.84		
					0.2-0.4 mm vs. Sham	0.9673	No	ns	-0.18	0.45	-0.80		
					0.4-0.6 mm vs. Sham	0.9996	No	ns	0.08	0.70	-0.55		
Supplementary Figure 4b<br>(Vm)	Unpaired t test<br>(two-tailed)	t = 1.353	0.1787	-	-	-	No	ns	1.298	-0.60	3.20	Yes (F test)	Yes (except Sham)
Supplementary Figure 4b<br>(Thre. curr.)	Unpaired t test<br>(two-tailed)	t = 0.1497	0.8816	-	-	-	No	ns	1.733	-21.55	25.02	Yes (F test)	Yes (except Sham)
Supplementary Figure 4c	Two-way ANOVA	F = 1.559	0.2123	Sidak's	Sham - GBM22 Contralateral							No	Yes (Sham+GBM22 Contralateral)<br>Some data set (Sham)
					0	>0.9999	No	ns	-0.05	5.63	-5.72		
					20	0.9613	No	ns	-2.19	3.48	-7.87		
					40	0.1393	No	ns	-4.93	0.74	-10.61		
					60	0.1022	No	ns	-5.17	0.51	-10.84		
					80	0.8559	No	ns	-2.73	2.94	-8.41		
					100	>0.9999	No	ns	-0.71	4.97	-6.38		
					120	>0.9999	No	ns	0.18	5.86	-5.49		
					140	0.9919	No	ns	1.77	7.44	-3.91		
					160	0.8902	No	ns	2.60	8.28	-3.07		
					180	0.6754	No	ns	3.27	8.94	-2.41		
Supplementary Figure 4d<br>(Vm)	Unpaired t test<br>(two-tailed)	t = 2.064	0.0634	-	-	-	No	ns	2.357	-0.16	4.87	Yes (F test)	n too small
Supplementary Figure 4d<br>(Thre. curr.)	Welch's t test<br>(two-tailed)	t = 0.4375	0.6726	-	-	-	No	ns	-7.417	-46.10	31.26	No	Yes (Sham)<br>n too small (GBM14)
Supplementary Figure 4d<br>(Rin)	Welch's t test<br>(two-tailed)	t = 1.298	0.2153	-	-	-	No	ns	47.68	-31.11	126.50	No	Yes (except Sham)
Supplementary Figure 4d<br>(Cm)	Welch's t test<br>(two-tailed)	t = 2.749	0.0106	-	-	-	Yes	*	-13.79	-24.08	-3.49	No	Yes (except GBM14)
Supplementary Figure 4e	Two-way ANOVA	F = 4.588	0.0338	Sidak's	Sham - GBM22 Contralateral							No (Sham)<br>Yes (Sham+GBM22 Contralateral)	Yes (Sham)<br>n too small (Sham+GBM22 Contralateral)
					0	>0.9999	No	ns	0.00	47.91	-47.91		
					20	>0.9999	No	ns	0.89	48.79	-47.02		
					40	0.9935	No	ns	-14.36	33.55	-62.26		
					60	>0.9999	No	ns	-7.97	39.94	-55.88		
					80	0.9215	No	ns	-20.56	27.35	-68.46		
					100	0.9584	No	ns	-18.57	29.34	-66.48		
					120	0.9936	No	ns	-14.34	33.56	-62.25		
					140	0.9985	No	ns	-11.96	35.95	-59.86		
					160	0.9968	No	ns	-13.16	34.75	-61.06		
					180	0.9941	No	ns	-14.17	33.74	-62.08		

Supplementary Figure 5a (Sham, 180 pA)	One-way ANOVA	F = 1.327	0.2762	Tukey's		-70 mV vs. -65 mV	0.7047	No	ns	-17.79	36.20	-71.79	Yes	Yes
						-70 mV vs. -70 mV	0.2449	No	ns	-36.20	17.79	-90.19		
						-65 mV vs. -70 mV	0.6878	No	ns	-18.41	35.59	-72.40		
Supplementary Figure 5a (GBM22)	One-way ANOVA	F = 0.2451	0.7901	Tukey's		-70 mV vs. -65 mV	0.7047	No	ns	-7.67	65.45	-80.79	Yes	n too small
						-70 mV vs. -70 mV	0.2449	No	ns	-16.67	56.45	-89.79		
						-65 mV vs. -70 mV	0.6878	No	ns	-9.00	64.12	-82.12		
Supplementary Figure 5a (Sham, 500 pA)	One-way ANOVA	F = 0.07246	0.9302	Tukey's		-70 mV vs. -65 mV	0.9757	No	ns	-4.63	48.41	-57.66	Yes	Yes
						-70 mV vs. -70 mV	0.9237	No	ns	-8.31	44.72	-61.34		
						-65 mV vs. -70 mV	0.9845	No	ns	-3.69	49.34	-56.72		
Supplementary Figure 5b	Two-way ANOVA	F = 122.8	<0.0001	Tukey's	0									
						Sham vs. GBM22	>0.9999	No	ns	-0.44	6.83	-7.72	No	Yes (about half of the groups)
						Sham vs. GBM22 (D-AP5+CNQX)	>0.9999	No	ns	0.00	7.28	-7.28		
						GBM22 vs. GBM22 (D-AP5+CNQX)	>0.9999	No	ns	0.44	8.23	-7.34		
					20									
						Sham vs. GBM22	0.8884	No	ns	-4.47	2.81	-11.75		
						Sham vs. GBM22 (D-AP5+CNQX)	>0.9999	No	ns	-0.21	7.07	-7.49		
						GBM22 vs. GBM22 (D-AP5+CNQX)	0.9686	No	ns	4.26	12.04	-3.52		
					40									
						Sham vs. GBM22	0.0017	Yes	**	-8.94	-1.66	-16.21		
						Sham vs. GBM22 (D-AP5+CNQX)	>0.9999	No	ns	-1.31	5.97	-8.58		
						GBM22 vs. GBM22 (D-AP5+CNQX)	0.0637	No	ns	7.63	15.41	-0.15		
					60									
						Sham vs. GBM22	<0.0001	Yes	****	-11.88	-4.60	-19.16		
						Sham vs. GBM22 (D-AP5+CNQX)	>0.9999	No	ns	-1.61	5.66	-8.89		
						GBM22 vs. GBM22 (D-AP5+CNQX)	0.0003	Yes	***	10.27	18.05	2.48		
					80									
						Sham vs. GBM22	<0.0001	Yes	****	-12.40	-5.12	-19.68		
						Sham vs. GBM22 (D-AP5+CNQX)	>0.9999	No	ns	-0.59	6.69	-7.87		
						GBM22 vs. GBM22 (D-AP5+CNQX)	<0.0001	Yes	****	11.81	19.59	4.03		
					100									
						Sham vs. GBM22	<0.0001	Yes	****	-11.86	-4.58	-19.14		
						Sham vs. GBM22 (D-AP5+CNQX)	>0.9999	No	ns	-0.17	7.11	-7.45		
						GBM22 vs. GBM22 (D-AP5+CNQX)	<0.0001	Yes	****	11.69	19.47	3.91		
					120									
						Sham vs. GBM22	<0.0001	Yes	****	-11.39	-4.11	-18.67		
						Sham vs. GBM22 (D-AP5+CNQX)	>0.9999	No	ns	-0.56	6.72	-7.83		
						GBM22 vs. GBM22 (D-AP5+CNQX)	<0.0001	Yes	****	10.83	18.62	3.05		
					140									
						Sham vs. GBM22	<0.0001	Yes	****	-10.84	-3.56	-18.12		
						Sham vs. GBM22 (D-AP5+CNQX)	>0.9999	No	ns	-0.53	6.75	-7.81		
						GBM22 vs. GBM22 (D-AP5+CNQX)	0.0003	Yes	***	10.31	18.10	2.53		
					160									
						Sham vs. GBM22	0.0009	Yes	***	-9.20	-1.92	-16.48		
						Sham vs. GBM22 (D-AP5+CNQX)	>0.9999	No	ns	-0.88	6.40	-8.16		
						GBM22 vs. GBM22 (D-AP5+CNQX)	0.0196	Yes	*	8.33	16.11	0.55		
					180									
						Sham vs. GBM22	0.0094	Yes	**	-8.16	-0.88	-15.44		
						Sham vs. GBM22 (D-AP5+CNQX)	>0.9999	No	ns	-1.57	5.71	-8.85		
						GBM22 vs. GBM22 (D-AP5+CNQX)	0.2611	No	ns	6.59	14.37	-1.20		
Supplementary Figure 5c	Two-way ANOVA	F = 104.1	<0.0001	Tukey's	0								No	Yes (about half of the groups)
						Sham vs. GBM22	>0.9999	No	ns	-0.11	48.57	-48.79		
						Sham vs. GBM22 (D-AP5+CNQX)	>0.9999	No	ns	0.00	49.19	-49.19		
						GBM22 vs. GBM22 (D-AP5+CNQX)	>0.9999	No	ns	0.11	41.85	-41.63		
					20									
						Sham vs. GBM22	>0.9999	No	ns	1.27	49.94	-47.41		
						Sham vs. GBM22 (D-AP5+CNQX)	>0.9999	No	ns	4.54	53.73	-44.64		
						GBM22 vs. GBM22 (D-AP5+CNQX)	>0.9999	No	ns	3.27	45.01	-38.47		
					40									
						Sham vs. GBM22	>0.9999	No	ns	4.06	52.73	-44.62		
						Sham vs. GBM22 (D-AP5+CNQX)	>0.9999	No	ns	14.03	63.22	-35.16		
						GBM22 vs. GBM22 (D-AP5+CNQX)	>0.9999	No	ns	9.97	51.71	-31.77		
					60									
						Sham vs. GBM22	0.9822	No	ns	25.32	74.00	-23.36		
						Sham vs. GBM22 (D-AP5+CNQX)	0.1345	No	ns	44.95	94.14	-4.23		
						GBM22 vs. GBM22 (D-AP5+CNQX)	0.9958	No	ns	19.63	61.37	-22.11		
					80									
						Sham vs. GBM22	0.2099	No	ns	42.30	90.98	-6.38		
						Sham vs. GBM22 (D-AP5+CNQX)	0.0002	Yes	***	66.12	115.31	16.94		
						GBM22 vs. GBM22 (D-AP5+CNQX)	0.9449	No	ns	23.82	65.56	-17.92		
					100									
						Sham vs. GBM22	0.0217	Yes	*	51.78	100.46	3.10		
						Sham vs. GBM22 (D-AP5+CNQX)	<0.0001	Yes	****	81.82	131.01	32.64		
						GBM22 vs. GBM22 (D-AP5+CNQX)	0.6143	No	ns	30.05	71.79	-11.69		
					120									
						Sham vs. GBM22	0.0002	Yes	***	66.58	115.26	17.90		
						Sham vs. GBM22 (D-AP5+CNQX)	<0.0001	Yes	****	90.39	139.57	41.20		
						GBM22 vs. GBM22 (D-AP5+CNQX)	0.9453	No	ns	23.81	65.55	-17.93		
					140									
						Sham vs. GBM22	<0.0001	Yes	****	76.46	125.13	27.78		

					Sham vs. GBM22 (D-AP5+CNQX)	<0.0001	Yes	****	97.84	147.03	48.66		
					GBM22 vs. GBM22 (D-AP5+CNQX)	0.9855	No	ns	21.39	63.13	-20.35		
					160								
					Sham vs. GBM22	<0.0001	Yes	****	86.87	135.54	38.19		
					Sham vs. GBM22 (D-AP5+CNQX)	<0.0001	Yes	****	95.70	144.89	46.51		
					GBM22 vs. GBM22 (D-AP5+CNQX)	>0.9999	No	ns	8.83	50.57	-32.91		
					180								
					Sham vs. GBM22	<0.0001	Yes	****	94.12	142.80	45.44		
					Sham vs. GBM22 (D-AP5+CNQX)	<0.0001	Yes	****	89.81	139.00	40.63		
					GBM22 vs. GBM22 (D-AP5+CNQX)	>0.9999	No	ns	-4.31	37.43	-46.05		
Supplementary Figure 6c (Vm)	Unpaired t test (two-tailed)	t = 2.29	0.0428	-	-	-	Yes	*	-4.024	-7.89	-0.16	Yes (F test)	n too small
Supplementary Figure 6c (Thre. curr.)	Unpaired t test (two-tailed)	t = 0.8664	0.402	-	-	-	No	ns	17.32	-25.87	60.51	Yes (F test)	Yes (Control)<br>n too small (ChABC)
Supplementary Figure 6c (Rin)	Welch's t test (two-tailed)	t = 0.6899	0.5008	-	-	-	No	ns	25.86	-53.99	105.70	No	Yes (except ChABC)
Supplementary Figure 6d (Vm)	Unpaired t test (two-tailed)	t = 1.507	0.1339	-	-	-	No	ns	-1.002	-2.32	0.31	Yes (F test)	Yes (except Control)
Supplementary Figure 6d (Thre. curr.)	Unpaired t test (two-tailed)	t = 0.6652	0.508	-	-	-	No	ns	-5.219	-20.86	10.42	Yes (F test)	Yes (except Control)
Supplementary Figure 6d (Rin)	Unpaired t test (two-tailed)	t = 0.7746	0.4421	-	-	-	No	ns	-19.5	-70.00	31.01	Yes (F test)	Yes (except Control)
Supplementary Figure 8b (ACSF, FSN)	Paired t test (two-tailed)	t = 0.9574	0.3589	-	-	-	No	ns	-1	-3.30	1.30	Yes (pairing effective)	Yes
Supplementary Figure 8b (ChABC, FSN)	Paired t test (two-tailed)	t = 2.57	0.0279	-	-	-	Yes	*	-2.909	-5.43	-0.39	No (pairing not effective)	Yes
Supplementary Figure 8b (ACSF, excitatory)	Paired t test (two-tailed)	t = 1.492	0.1862	-	-	-	No	ns	1.714	-1.10	4.53	Yes (pairing effective)	n too small
Supplementary Figure 8b (ChABC, excitatory)	Paired t test (two-tailed)	t = 4.742	0.0051	-	-	-	Yes	**	-11.33	-17.48	-5.19	No (pairing not effective)	n too small
Supplementary Figure 8c (Rin, ACSF, excitatory)	Paired t test (two-tailed)	t = 0.01177	0.991	-	-	-	No	ns	-0.176	-36.77	36.41	No (pairing not effective)	n too small
Supplementary Figure 8c (Rin, ChABC, excitatory)	Paired t test (two-tailed)	t = 0.08278	0.9372	-	-	-	No	ns	1.891	-56.82	60.60	No (pairing not effective)	n too small
Supplementary Figure 8c (Cm, ACSF, excitatory)	Paired t test (two-tailed)	t = 0.8622	0.4217	-	-	-	No	ns	-4.873	-18.70	8.96	Yes	n too small
Supplementary Figure 8c (Cm, ChABC, excitatory)	Paired t test (two-tailed)	t = 0.7646	0.479	-	-	-	No	ns	-10.02	-43.71	23.67	No (pairing not effective)	n too small
